# Sulfonylimide-Based Single-Ion-Conducting Porous Organic Polymer Electrolytes for Enhanced Performance of Solid-State Lithium Batteries

**DOI:** 10.1021/acsami.5c20914

**Published:** 2026-02-10

**Authors:** Pin-Jyun Chen, Jaturon Kumchompoo, Bo-Lin Chen, Yun-Chen Chuang, Bei-Chun Liao, Chia-Chen Li, Jyh-Tsung Lee

**Affiliations:** † Department of Chemistry, 34874 National Sun Yat-sen University, Kaohsiung 80424, Taiwan; ‡ Department of Chemistry, Faculty of Science and Technology, Thammasat University, Pathumtani 12120, Thailand; § Department of Materials Science and Engineering, 34881 National Tsing Hua University, Hsinchu 300044, Taiwan; ∥ Department of Medicinal and Applied Chemistry, Kaohsiung Medical University, Kaohsiung 80708, Taiwan

**Keywords:** single-ion conductor, porous organic polymer, solid polymer electrolyte, sulfonylimide, solid electrolyte interphase, cathode electrolyte interphase

## Abstract

The development of solid-state electrolytes with high ionic conductivity and interfacial stability is vital for next-generation lithium-ion batteries. Concentration polarization in conventional electrolytes accelerates solid electrolyte interphase (SEI) and cathode electrolyte interphase (CEI) growth, thereby limiting cycle life and reliability. Here, we report a lithium sulfonylimide-based single-ion-conducting porous organic polymer (Li-SSP) electrolyte designed to suppress anion mobility and enhance Li^+^ transport. The Li-SSP was synthesized via Sonogashira coupling of 4-bromo-*N*-((4-aminophenyl)­sulfonyl)­benzenesulfonamide with 1,3,5-triethynylbenzene. Fourier-transform infrared spectroscopy confirmed its chemical structure, while Brunauer–Emmett–Teller analysis revealed coexisting mesoporous and microporous architectures with a high surface area of 271 m^2^ g^–1^. The immobilized anionic groups in the porous framework provide continuous Li^+^ conduction pathways, effectively reducing concentration polarization and improving electrochemical stability. The lithium bis­(trifluoromethanesulfonyl)­imide (LiTFSI)/poly­(vinylidene fluoride-*co*-hexafluoropropylene) (PVDF-HFP)/Li-SSP composite electrolyte exhibits a high ionic conductivity of 4.04 × 10^–4^ S cm^–1^ at 30 °C and a high Li^+^ transference number of 0.70, significantly higher than that of LiTFSI/PVDF-HFP electrolytes (0.18). A Li||Li symmetric cell demonstrates stable Li plating/stripping for over 400 h with low overpotential, confirming excellent interfacial compatibility. A LiFePO_4_ half-cell with the Li-SSP composite electrolyte delivers high capacities of 148.1 mAh g^–1^ at 0.2 C and 86.7 mAh g^–1^ at 5 C, along with 96.1% capacity retention after 300 cycles at 0.5 C. Scanning electron microscopy, transmission electron microscopy, and X-ray photoelectron spectroscopy analyses further confirm the formation of thinner and chemically stable CEI films, resulting in reduced interfacial resistance. These results highlight that rational design of Li-SSP electrolytes enables high Li^+^ transference, robust interfacial stability, and extended cycle life, offering a promising pathway toward safe and durable solid-state lithium-ion batteries.

## Graphical abstract

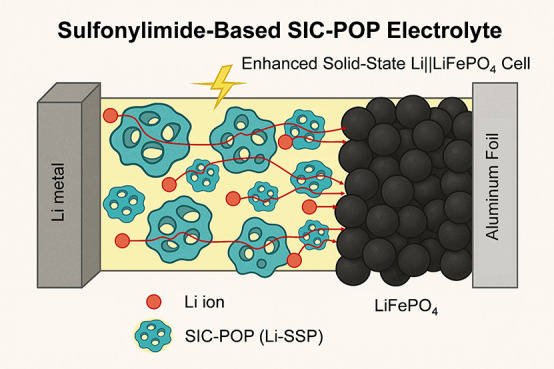

## 1 Introduction

Along with the development of technology, lithium-ion batteries are one of the widely used energy storage systems for portable electronic devices and electric vehicles. However, safety concerns have been a big issue for lithium-ion batteries.^1–3^ The development of high-performance solid electrolytes has emerged as a cornerstone for advancing next-generation batteries, particularly solid-state lithium batteries, which offer improved safety and higher energy density compared to conventional liquid electrolyte lithium-ion batteries.^4–6^

Solid polymer electrolytes have gained considerable attention in recent years due to their high mechanical flexibility, which facilitates better interfacial contact and reduces interfacial resistance.^7,8^ However, the main drawback lies in the significantly low ionic conductivity (∼10^–7^ to 10^–5^ S cm^–1^ at room temperature), which requires elevated temperatures to reach practically usable levels.^9,10^ In addition, conventional solid polymer electrolytes contain dual ion lithium salt(s) and polymer matrix. The lithium salt would be dissociated into lithium ions and anions to migrate through the polymer matrix. With a lithium-ion transference number (*t*_Li^+^_) of around 0.2–0.4, this leads to severe concentration polarization.^11–13^ This effect can accelerate lithium dendrite growth, increase the resistance of batteries, and decompose anion to form thick cathode electrolyte interphase (CEI) films, thus limiting cycle life and reliability of polymer-based solid-state batteries.^14^

Single-ion conducting (SIC) polymers have attracted increasing attention because their high *t*_Li^+^_ suppresses concentration polarization in electrolytes, thereby enhancing battery performance. These polymers are typically designed with backbones bearing bulky anionic groups, which immobilize the anions and facilitate lithium-ion transport.^15–18^ In addition to mitigating concentration polarization, SIC polymers reduce lithium salt depletion and promote uniform Li^+^ flux, minimizing current density spikes and enabling smooth, homogeneous lithium deposition on the anode surface. This effectively suppresses dendrite nucleation and growth.^19–21^ Reported anionic groups in SIC polymers include carboxylate, sulfonate, boronate, and sulfonylimide moieties.^18^ Among these, sulfonylimide-based systems are particularly promising. In this structure, the nitrogen atom is bonded to two strongly electron-withdrawing sulfonyl (−SO_2_−) groups, which enhances the ionic character of the N–Li bond. Consequently, lithium ions can be more readily solvated within the polymer matrix, leading to high ionic conductivity. Furthermore, the sulfonyl group provides a wide electrochemical stability window and versatile opportunities for molecular design.^18^ Thus, sulfonylimide-based SIC polymers are considered among the most promising candidates for practical applications.

In recent years, porous organic polymers (POPs) have attracted considerable attention owing to their tunable structures, high surface area, and interconnected microporous networks.^22–25^ Moreover, POPs with abundant micropores and extended frameworks offer high stability and continuous channels for rapid lithium-ion transport.^26–29^ To further enhance their electrochemical properties, SIC polymers have been incorporated into POP-based matrices such as poly­(vinylidene fluoride-*co*-hexafluoropropylene) (PVDF-HFP),^30–32^ poly­(ethylene oxide) (PEO),^33,34^ and poly­(vinylidene fluoride) (PVDF).^35,36^ This integration reduces concentration polarization, increases *t*_Li^+^_, and thereby improves cycle-life performance.^20,37^ At the same time, these composite polymer electrolytes present a promising alternative to inorganic–polymer hybrids by overcoming particle aggregation and interfacial incompatibility issues typically associated with inorganic fillers.^37,38^

In this study, we synthesized a lithium sulfonylimide-based SIC-POP (Li-SSP), as a solid polymer electrolyte for solid-state lithium batteries. In this system, sulfonylimide anions are immobilized within the POP framework, while the delocalization and strong electron-withdrawing effect of the two sulfonyl groups enhance lithium-ion dissociation efficiency. The Li-SSP was prepared via a Sonogashira coupling reaction between the terminal alkynes of 1,3,5-triethynylbenzene and the phenyl bromide groups of lithium ((4-((4-bromophenyl)­sulfonamido)­phenyl)­sulfonyl)­((4-bromophenyl)­sulfonyl)­amide (Li-BPSSA), yielding a highly cross-linked structure. The ionic conductivity and electrochemical stability of a composite electrolyte composed of lithium bis­(trifluoromethanesulfonyl)­imide (LiTFSI), PVDF-HFP, and Li-SSP were systematically investigated. Furthermore, the electrochemical performance of Li|LiFePO_4_ (LFP) cells employing this electrolyte was evaluated by electrochemical impedance spectroscopy (EIS), distribution of relaxation time (DRT) analysis, galvanostatic intermittent titration technique (GITT), and cell cycling tests. Surface characterization by scanning electron microscopy (SEM), transmission electron microscopy (TEM), and X-ray photoelectron spectroscopy (XPS) confirmed that the LiTFSI/PVDF-HFP/Li-SSP composite electrolyte facilitates the formation of thinner and more stable CEI films on the LFP cathode. This work demonstrates that integrating the Li-SSP into conventional polymer electrolytes is an effective strategy to achieve high ionic conductivity, improved interfacial stability, and enhanced cycling performance, highlighting its potential as a promising electrolyte design for next-generation solid-state lithium batteries.

## 2 Experimental Section

### 2.1 Materials

4-Nitrobenzenesulfonamide (98%), 4-bromobenzenesulfonyl chloride (98%), tin­(II) chloride dihydrate (98%), copper­(I) iodide (CuI, 99%), and tetrakis­(triphenylphosphine)­palladium(0) (Pd­(PPh_3_)_4_, 99%) were purchased from Nova-Matls. 4-Dimethylaminopyridine (DMAP, 99%) and lithium perchlorate (LiClO_4_, 95%) were purchased from Alfa Aesar. 1,3,5-Tribromobenzene (98%) was purchased from TCI. 2-Methyl-3-butyn-2-ol (98%), *n*-butyl lithium (*n*-BuLi, 2.0 M in cyclohexane), hydrochloric acid (HCl, ≥ 37%), triethylamine (≥99.5%), and poly­(vinylidene fluoride-*co*-hexafluoropropylene) (PVDF-HFP, *M*_*w*_ = 400,000) were purchased from Sigma-Aldrich. Potassium hydroxide (KOH, 85%), ethyl acetate (GR grade), *n*-hexane (EP grade), acetone (GR grade), *N,N*-dimethylformamide (DMF, 99.5%, GR grade), and dichloromethane (99.5%, GR grade) were purchased from Duksan. Sodium hydrogen carbonate (NaHCO_3_, 99.5%) was purchased from SHOWA. Diethyl ether (≥99.0%) and toluene (≥99.0%) were purchased from ECHO. Acetonitrile (HPLC grade) was purchased from JT Baker. *N*-Methyl-2-pyrrolidone (NMP, 99%) was purchased from Janssen. Lithium bis­(trifluoromethanesulfonyl)­imide (LiTFSI, 99%) was purchased from Acros. Ethylene carbonate (EC), propylene carbonate (PC), diethyl carbonate (DEC), and fluoroethylene carbonate (FEC) were purchased from Novolyte Technologies. Super P was purchased from TIMCAL. Poly­(vinylidene fluoride) (PVDF, KF-1100) was purchased from Kureha. All chemicals were used as received.

### 2.2 Synthesis of 4-Bromo-*N*-((4-nitrophenyl)­sulfonyl)­benzenesulfonamide (BNBSA)

4-Bromobenzenesulfonyl chloride (2 g, 7.83 mmol), 4-nitrobenzenesulfonamide (1.9 g, 9.39 mmol), and DMAP (95 mg, 0.78 mmol) were added in a dried Schlenk flask. Then, triethylamine (1.2 mL, 8.61 mmol) and anhydrous acetonitrile (50 mL) were added to the above flask and stirred for 12 h under a nitrogen atmosphere. After the reaction, the precipitate was separated from the solution and the solution was concentrated with a rotary evaporator to give a white solid. Dissolved the white solid compound with dichloromethane and extracted with 1.0 M HCl_(aq)_ (10 mL) three times. The organic layer was separated from the aqueous phase. After drying over anhydrous magnesium sulfate, the mixture was filtered through a suction, and the filtrate was removed by rotary evaporation to give BNBSA (63% yield). ^1^H NMR (300 MHz, deuterium oxide) δ 8.11 (d, *J* = 9.1 Hz, 2H), 7.65 (d, *J* = 9.1 Hz, 2H), 7.42 (d, *J* = 8.9 Hz, 2H), 7.34 (d, *J* = 8.9 Hz, 2H). ^13^C NMR (101 MHz, DMSO-*d*_6_) δ 131.5, 128.9, 128.3, 123.9, 40.7, 40.4, 40.2, 40.0, 39.8, 39.6, 39.4. HRMS (ESI-Orbitrap): *m*/*z* calcd for C_12_H_8_O_6_N_2_^79^Br^32^S_2_ (M^+^): 418.9013; found 418.9002.

### 2.3 Synthesis of 4-Bromo-*N*-((4-aminophenyl)­sulfonyl)­benzenesulfonamide (BABSA)

BNBSA (2 g, 4.74 mmol) was dissolved with dichloromethane (28 mL) in a 100 mL flask and then stirred at room temperature. Meanwhile, tin­(II) chloride dihydrate (3.22 g, 14.24 mmol) was dissolved with methanol (7 mL) in a 20 mL beaker. When it was completely dissolved, treated with concentrated hydrochloric acid (0.7 mL). Then, the methanol solution was added dropwise into the above dichloromethane solution of BNBSA. The solution was stirred at 40 °C for 4 h to give a white precipitate. The white precipitate was collected through filtration and washed with a mixture solvent of dichloromethane/methanol (= 3/1, by volume, 40 mL) three times, and then dried in a vacuum oven at 50 °C for 12 h to obtain a pure BABSA. (88% yield) ^1^H NMR (300 MHz, DMSO-*d*_6_) δ 7.68–7.51 (m, 6H), 6.95 (d, *J* = 8.6 Hz, 2H). ^13^C NMR (101 MHz, DMSO-*d*_6_) δ 145.3, 141.6, 139.2, 131.6, 128.9, 128.6, 124.7, 120.4. HRMS (ESI-Orbitrap): *m*/*z* calcd for C_12_H_10_O_4_N_2_^79^Br^32^S_2_ (M^+^): 388.9271; found 388.9261.

### 2.4 Synthesis of Lithium ((4-((4-Bromophenyl)­sulfonamido)­phenyl)­sulfonyl)­((4-bromophenyl)­sulfonyl)­amide (Li-BPSSA)

BABSA (2 g, 5.07 mmol), 4-bromobenzenesulfonyl chloride (1.6 g, 6.25 mmol), and DMAP (76 mg, 0.62 mmol) were added in a dried Schlenk flask. Then, triethylamine (0.725 mL, 5.2 mmol) and anhydrous acetonitrile (50 mL) were added to a flask and stirred for 12 h under a nitrogen atmosphere. After the reaction, the mixture was filtered to collect the filtrate, and the solvent was removed by a rotary evaporator to give a light orange solid. Then, the solid was dissolved by dichloromethane (10 mL) and extracted with 4 wt % NaHCO_3(aq)_ (20 mL) three times and 1.0 M HCl_(aq)_ (20 mL) twice. Then, 1.5 M K_2_CO_3(aq)_ (50 mL) was added to the organic layer, yielding a pale-yellow solid. The precipitate was filtrate through a suction, dried, and then transferred into a round-bottom flask, followed by addition of dry acetonitrile (100 mL) and LiClO_4_ (0.54, 5.07 mmol). The mixture was refluxed under a nitrogen atmosphere for 12 h. After the reaction, KClO_4_ was removed by suction filtration, and the solvent was removed by rotary evaporation. The resulting solid was dissolved in a mixture solvent of toluene and acetonitrile (4:1 by volume, 50 mL) and reacted at 60 °C for 6 h. After the reaction, the mixture was filtrated through a suction, and the filtrate was removed by rotary evaporation, and then dried in a vacuum oven at 70 °C for 12 h to obtain Li-BPSSA (76% yield). ^1^H NMR (300 MHz, DMSO-*d*_6_) δ 7.64 (d, *J* = 8.4 Hz, 2H), 7.58–7.45 (m, 6H), 7.18 (d, *J* = 8.7 Hz, 2H), 6.66 (d, *J* = 8.7 Hz, 2H). ^13^C NMR (101 MHz, DMSO-*d*_6_) δ 147.3, 131.4, 131.1, 128.8, 127.0, 119.2, 40.7, 40.5, 40.3, 40.0, 39.9, 39.6, 39.4. HRMS (ESI-Orbitrap): *m*/*z* calcd for C_18_H_13_O_6_N_2_^79^Br_2_^32^S_3_ (M^+^): 606.8308; found 606.8305.

### 2.5 Synthesis of 1,3,5-Tris­(3-methyl-3-hydroxybut-1-ynyl)­benzene

A mixture of 1,3,5-tribromobenzene (1 g, 6 mmol), 2-methylbut-3-yn-2-ol (1.2 g, 28 mmol), CuI (51 mg, 0.5 mmol), and Pd­(PPh_3_)_4_ (175 mg, 0.25 mmol) was added in a two-neck round-bottom flask equipped with a reflux condenser. The system was evacuated and backfilled with nitrogen to maintain an inert atmosphere. Triethylamine (15 mL) was degassed with nitrogen for 1 min and then added to the reaction flask. The mixture was refluxed for 5 h under a nitrogen atmosphere. After completion of the reaction, the mixture was filtered and the residue was washed with diethyl ether. The combined filtrate was concentrated under reduced pressure to remove excess solvent, affording a crude product. The product was purified by column chromatography on silica gel using ethyl acetate/*n*-hexane (1:1, v/v) as the eluent to give a yellow solid in 83% yield. ^1^H NMR (300 MHz, chloroform-*d*) δ 7.42 (s, 3H), 1.62 (s, 18H). ^13^C NMR (101 MHz, chloroform-*d*) δ 134.3, 123.4, 95.0, 80.6, 65.6, 31.5.

### 2.6 Synthesis of 1,3,5-Triethynylbenzene

A mixture of 1,3,5-tris­(3-methyl-3-hydroxybut-1-ynyl)­benzene (1 g, 3.08 mmol), KOH (604 mg, 10.78 mmol), and toluene (10 mL) was added to a single-neck round-bottom flask and stirred. The reaction mixture was heated under reflux while monitoring the progress by thin-layer chromatography (TLC) until complete consumption of the starting material, 1,3,5-tris­(3-methyl-3-hydroxybut-1-ynyl)­benzene. After completion, the reaction mixture was filtered to remove residual KOH, and the filtrate was concentrated under reduced pressure to remove excess solvent, affording a crude product. The product was purified by column chromatography on silica gel using *n*-hexane as the eluent to give a white solid in 83% yield. ^1^H NMR (300 MHz, chloroform-*d*) δ 7.60 (s, 3H), 3.14 (s, 3H). ^13^C NMR (101 MHz, chloroform-*d*) δ 135.66, 122.93, 81.64, 78.73.

### 2.7 Synthesis of Li-SSP

Li**-**BPSSA (1460 mg, 2.25 mmol), triethynylbenzene (225 mg, 1.5 mmol), CuI (171 mg, 0.90 mmol), Pd­(PPh_3_)_4_ (520 mg, 0.45 mmol), anhydrous triethylamine (22.5 mL), and anhydrous DMF (75 mL) were added into a Schlenk flask. After three freeze–pump–thaw cycles, the mixture of the Schlenk flask was reacted at 100 °C for 72 h, resulting in a brown precipitate. After reaction, the precipitate was collected through the filtration and washed with DMF (50 mL) and methanol (50 mL) three times, respectively. The filtered precipitate was dried in a vacuum oven at 70 °C for 12 h to give SSP. After dried, the porous polymer SSP and anhydrous *n*-hexane (30 mL) were added into a dried Schlenk flask under a nitrogen atmosphere. Then, *n*-BuLi (2.0 M, 2 mL) was added into the flask dropwise and stirred at room temperature for 24 h to make the sulfonyl amide into lithium sulfonyl amide. After the reaction, the precipitate was collected by filtration and dried in a vacuum oven at 80 °C to get a single-ion-conductor of Li-SSP.

### 2.8 Fabrication of Solid-State Electrolytes

The LiTFSI/PVDF-HFP/Li-SSP electrolyte was composed of Li-SSP, PVDF-HFP, and LiTFSI (= 65:20:15, by weight). A LiTFSI/PVDF-HFP electrolyte as a control electrolyte (without Li-SSP) was composed of PVDF-HFP and LiTFSI (= 85:15, by weight). According to the above ratio, PVDF-HFP (1.018 g) and acetone (8 mL) were added in a vial, and then Li-SSP (0.312 g) and LiTFSI (0.234 g) (for the LiTFSI/PVDF-HFP electrolyte, only LiTFSI was added) were added into the PVDF-HFP solution and stirred at room temperature for 24 h. The mixture was coated onto an aluminum foil by a doctor blade with a thickness of 80 μm. The polymer electrolyte films were dried in a vacuum oven at 60 °C for 24 h. Finally, the polymer film was cut into circular pieces with a diameter of 18 mm and stored in an argon-filled glovebox.

### 2.9 Fabrication of LFP Electrodes

The LFP cathode was composed of LFP, Super P, and PVDF (80:15:5 by weight). According to the above ratio, PVDF (0.150 g) was dissolved in NMP (3 mL), and then LFP (2.4 g) and Super P (0.450 g) were added in the NMP solution of PVDF and was ball-milled homogeneously to give a slurry of cathode. The prepared slurry was coated onto aluminum foil by a doctor blade and subsequently dried in a vacuum oven at 90 °C for 12 h. The dried cathode is then calendered using a roller ball mill and cut into circular pieces with a diameter of 13 mm, of which the average thickness was 40 ± 5 μm, and the average mass loading of the LFP electrode was 0.9 mg cm^–2^.

### 2.10 Material Characterization

^1^H Nuclear magnetic resonance (^1^H NMR) spectra were recorded on a Bruker Avance-300 NMR spectrometer using a deuterated reagent as a solvent with tetramethylsilane as an internal reference. ^13^C NMR spectra were recorded on a JEOL-400 NMR spectrometer and using deuterated solvent as a solvent. Fourier-transform infrared spectroscopy (FTIR) spectra were recorded by an attenuated total reflection (ATR)-FTIR on a PerkinElmer UATR within a wavenumber range of 4000–400 cm^–1^ in 8 scans to characterize the functional groups of organic compounds and polymers. Inductively coupled plasma–mass spectrometry (ICP–MS) analysis to quantify the Pd content in Li-SSP was performed using a Thermo Fisher Scientific iCAP TQ instrument (Germany). Brunauer–Emmett–Teller (BET) analysis was recorded on ASAP 2020MP PLUS to characterize the pore size and surface area of the polymer. Prior to measurement, the samples were activated at 100 °C under vacuum for 6 h. SEM images were obtained using a ZEISS SUPER 55-VP instrument; TEM images were obtained using a Tecnai F20 G2MAT S-TWIN instrument. XPS spectra were measured on a PHI Quantera II. The high-resolution mass spectra (HRMS) of each compound were measured on a Thermo Fisher orbitrap Exploris 120 instrument. Nanoindentation measurements of the composite electrolytes were conducted at room temperature using a Keysight Technologies MTS nanoindenter, with the maximum indentation depth set to 300 nm. Thermogravimetric analysis (TGA) was performed using a METTLER TOLEDO TGA 1 STARe system. The measurements were conducted under a nitrogen atmosphere with a heating rate of 10 °C min^–1^, over a temperature range from room temperature to 900 °C.

### 2.11 Electrochemical Measurements

CR2032 coin cells were assembled to study the electrochemical properties of the Li|LFP cells with different electrolytes. The electrolytes were LiTFSI/PVDF-HFP and LiTFSI/PVDF-HFP with SIC-POP. The electrolyte membranes, which also served as separators, were wetted with 0.05 mL of EC/PC (1:1, v/v) to improve interfacial contact between the electrolyte and the electrodes. For ionic conductivity measurement, a symmetrical cell with stainless steel (SS) as electrodes were assembled and measured from 30 to 70 °C, using EIS measurements to obtain the resistance of electrolytes. For the *t*_Li^+^_ analysis, a symmetrical cell with lithium metal electrodes between electrolytes was measured at 30 °C to obtain the initial and steady-state current and resistance, then calculated by the Bruce-Vincent-Evans equation. For the linear sweep voltammetry (LSV) measurements, the scan range was from open circuit potential to 7.0 V using stainless steel as a working electrode and the lithium metal as the counter and reference electrodes. For the cyclic voltammetry (CV), the LFP electrode was used as the working electrode, and lithium foil was used as the counter and reference electrodes. The CV measurements were performed between a voltage range of 2.4–4.2 V at a scan rate of 0.1 mV s^–1^ on an electrochemical workstation (CHI 627d). For cell performance, the lithium electrode was used as the anode, and LFP was used as the cathode. The coin cells were discharged to 2.4 V and charged to 4.2 V by the LANDT battery test system CT3002A. EIS measurements were carried out under a frequency between 0.1 and 100000 Hz at a voltage of 3.4 V with a perturbation amplitude of 10 mV on an electrochemical workstation (CHI 750A). GITT measurements were carried out using Li|LFP cells with LiTFSI/PVDF-HFP and LiTFSI/PVDF-HFP/SIC-POP electrolytes. The measurements were performed within a voltage window of 2.4–4.2 V at a current rate of 0.2 C. Each galvanostatic step consisted of a 20 min charge/discharge pulse followed by a 10 min relaxation period, and this procedure was repeated until the cell voltage reached the specified limits. A total of 20 cycles were performed at a controlled temperature of 30 °C. DRT analysis was performed by deconvoluting the EIS data into the relaxation time distribution function, g­(τ). The relaxation time (τ) is related to the impedance frequency (f) by τ = 1/(2πf). This approach enables the deconvolution of overlapping electrochemical processes occurring over different time scales, providing deeper insight into the respective contributions of SEI (or CEI) layer formation and ionic conduction to the overall cell impedance. Lithium plating/stripping tests were performed using lithium metal as the working electrode and another lithium metal foil as both the reference and counter electrodes. The capacity was set to 0.5 mAh cm^–2^ with an electrode area of 2 cm^2^. The applied current was 1 mA, corresponding to a charge/discharge duration of 1 h for each half-cycle. All measurements were carried out under a controlled temperature of 30 °C.

## 3 Results and Discussion

### 3.1 Synthesis and Characterization of Li-SSP

In this study, the SIC-POP, Li-SSP, was synthesized via Sonogashira coupling, using triethynylbenzene as the core and Li-BPSSA as the linker to construct a macrocyclic polymer (Figure 1). The resulting macrocyclic network contains fixed sulfonylimide anions and enables Li^+^ conduction through interconnected channels. Figure 2 shows the synthesis of the SIC-POP electrolyte, Li-SSP. The synthetic route began with the reaction of 4-nitrobenzenesulfonamide and 4-bromobenzenesulfonyl chloride to give BABSA in 88% yield. BABSA was subsequently reduced with SnCl_2_ to generate the corresponding amine derivative. The primary amine group then reacted with the benzenesulfonyl chloride moiety of 4-bromobenzenesulfonyl chloride, producing a sulfonamide intermediate. Treatment of this intermediate with an acetonitrile solution of LiClO_4_ afforded the Li-BPSSA linker. Finally, Li-BPSSA was coupled with a triethynylbenzene core via a Sonogashira coupling reaction to form a POP framework, which subsequently reacted with *n*-butyllithium to yield the porous polymer Li-SSP.

**Figure 1:**
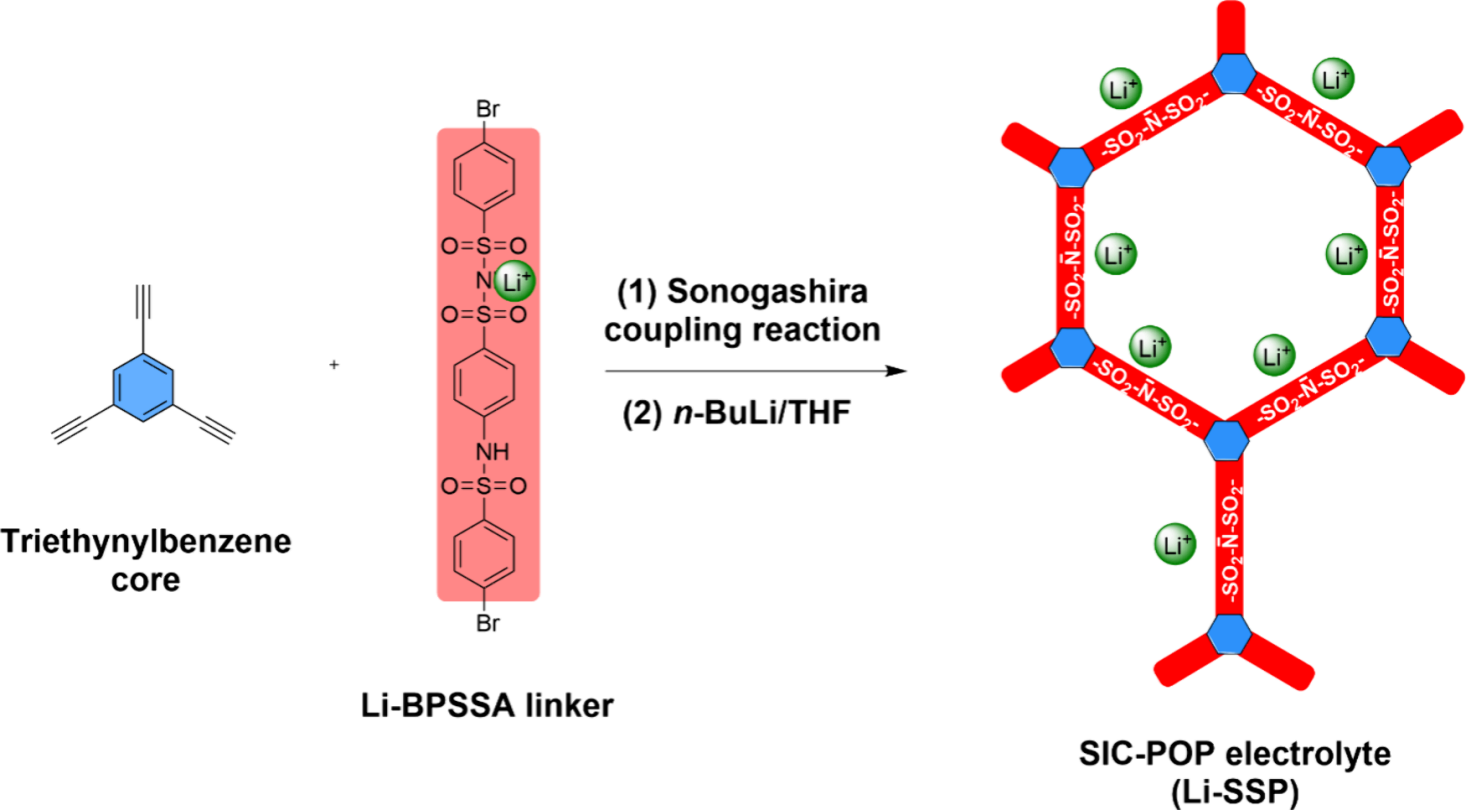
Schematic illustration of the synthesis of the single-ion-conducting porous organic polymer (SIC-POP, Li-SSP) via the Sonogashira coupling reaction between the triethynylbenzene core and the Li-BPSSA linker, and the Li^+^ conduction pathways within the resulting polymer network.

**Figure 2:**
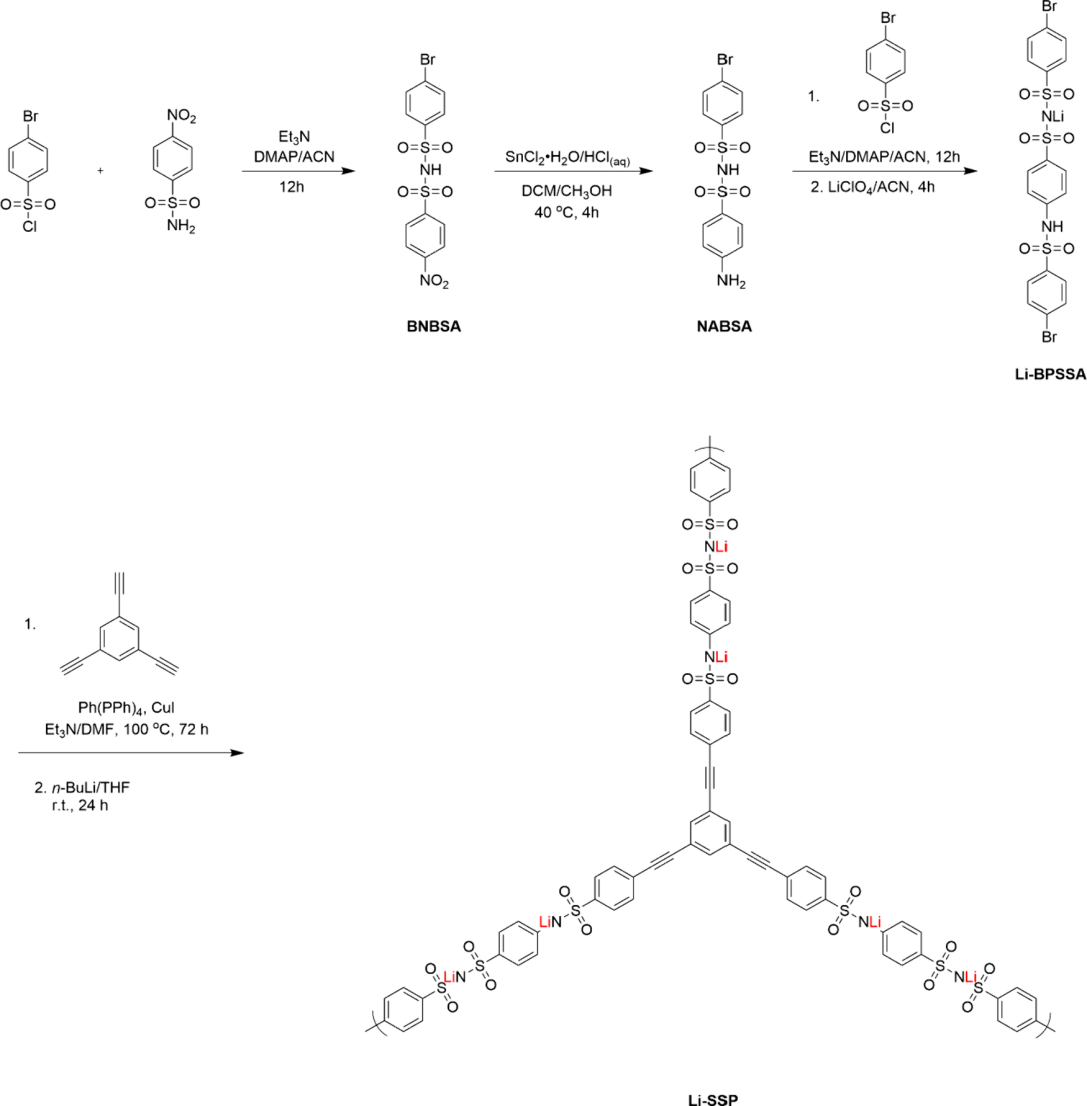
Synthesis of Li-SSP.

The chemical structure of Li-SSP was confirmed by IR spectroscopy. Figure 3a­(i)–(iii) shows the IR spectra of triethynylbenzene, Li-BPSSA, and Li-SSP, respectively. In Figure 3a­(i), the spectrum of the triethynylbenzene core exhibits characteristic absorption bands at 3277 cm^–1^ and 2109 cm^–1^, corresponding to terminal alkynyl C–H stretching and C≡C stretching vibrations, respectively.^39,40^ The IR spectrum of the Li-BPSSA linker in Figure 3a­(ii) shows absorption bands at 1493, 1268, 1147, and 557 cm^–1^, assigned to SO_2_ asymmetric stretching, SO_2_ symmetric stretching, N–S stretching, and SO_2_ bending, respectively.^41–43^ After polymerization, the IR spectrum of Li-SSP (Figure 3a­(iii)) no longer shows the C–Br stretching at 769 cm^–1^, nor the terminal alkynyl C–H stretching at 3277 cm^–1^ and C≡C stretching at 2109 cm^–1^. These spectral changes confirm that Li-BPSSA and triethynylbenzene successfully underwent Sonogashira coupling to yield Li-SSP, a single-ion-conducting POP.

**Figure 3:**
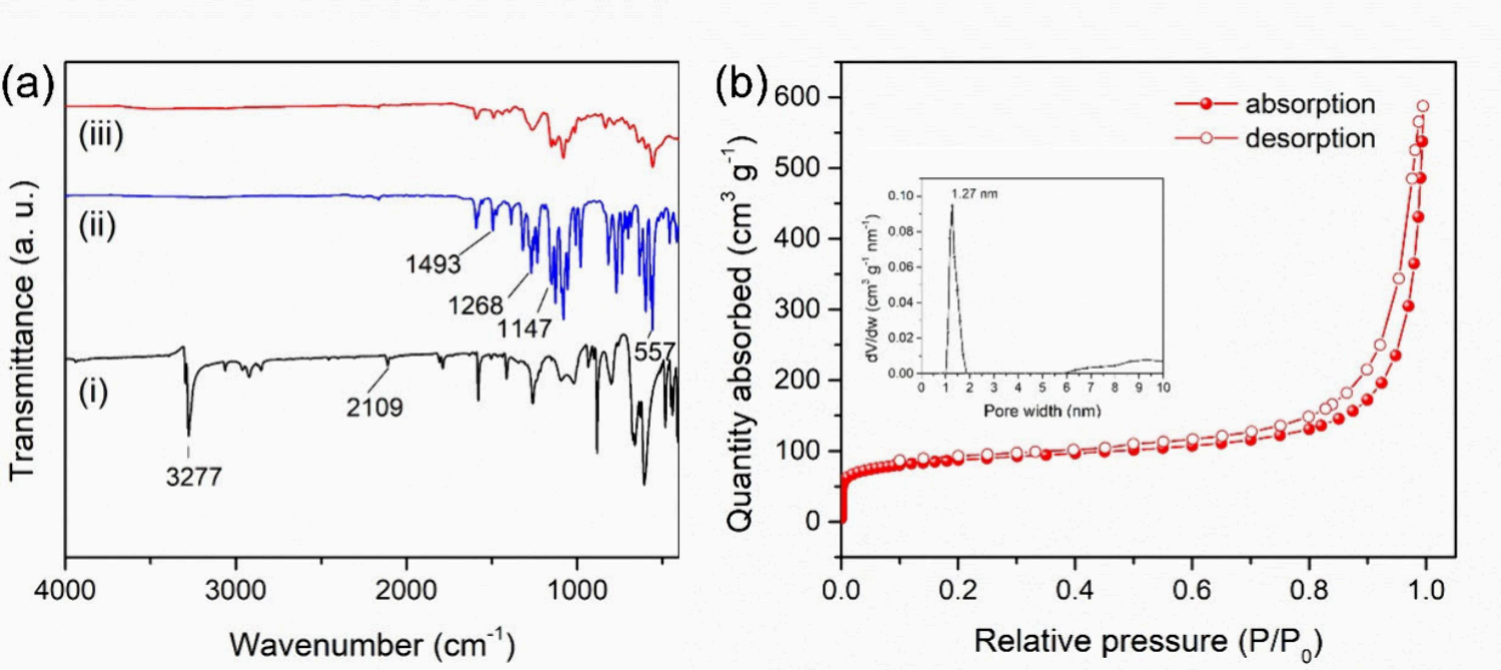
(a) IR spectra of (i) triethynylbenzene, (ii) Li-BPSSA, and (iii) Li-SSP. (b) BET surface area measurement of absorption (solid) and desorption (hole) for Li-SSP.

POPs are insoluble, highly cross-linked, and intrinsically microporous; therefore, the Pd residues are physically trapped within the polymer matrix. To verify the presence of Pd in Li-SSP, ICP–MS analysis was conducted, revealing a Pd content of 2.183 wt %, which is comparable to values reported for other Sonogashira-derived POP materials.^44^ Importantly, Li-SSP neither dissolves nor migrates during battery operation. As a result, the immobilized Pd residues are unlikely to participate in Li plating/stripping reactions and should not interfere with the electrochemical performance of the Li|LFP cells incorporating the Li-SSP electrolyte.

The BET surface area and pore characteristics of Li-SSP were determined by nitrogen adsorption–desorption analysis. Figure 3b shows the nitrogen adsorption–desorption isotherm of Li-SSP, which corresponds to a typical type IV isotherm with an H3-type hysteresis loop, indicating the coexistence of mesoporous and microporous structures. The steep uptake at P/P_o_ < 0.1 indicates the presence of micropores, while the pronounced increase in adsorption at P/P_o_ > 0.9 is attributed to interparticle voids or slit-like pores between lamellar aggregates. The Li-SSP exhibits a calculated BET surface area of 271 m^2^ g^–1^ and an average pore diameter of 1.27 nm (inset of Figure 3b), values that are consistent with those typically observed for POP materials.^22^ Such hierarchical porosity is expected to facilitate electrolyte infiltration and enhance lithium-ion transport within the SIC-POP electrolyte.

The TGA result (Figure S14) shows that the Li-SSP exhibits no detectable weight loss below 100 °C, indicating the absence of residual moisture or volatile species and confirming the excellent thermal stability of the material at temperatures relevant to battery operation. A gradual mass loss of approximately 6 wt % is observed between 100 and 300 °C. This early stage weight loss is typically attributed to the release of physically adsorbed water, trapped solvent molecules, or low-molecular-weight species within the porous polymer matrix, rather than the decomposition of the sulfonylimide functional groups. Prior studies have demonstrated that sulfonimide/sulfonylimide moieties possess high intrinsic thermal stability, with structural degradation generally occurring above 250–350 °C.^45^ Therefore, the Li-SSP maintains excellent thermal stability across the entire operational range of solid-state lithium batteries.

### 3.2 Electrochemical Properties

To evaluate the electrochemical stability of the Li-SSP composite electrolyte, LSV was conducted. As shown in Figure 4a­(i), the onset of oxidative current for the LiTFSI/PVDF-HFP electrolyte appears at 5.55 V vs Li/Li^+^. In contrast, the onset for the LiTFSI/PVDF-HFP/Li-SSP electrolyte shifts to 5.93 V (Figure 4a­(ii)), indicating that the incorporation of the Li-SSP single-ion conductor enhances interfacial stability. This improved stability highlights the potential of the Li-SSP composite electrolyte for application in Li|LFP solid-state lithium batteries.

**Figure 4:**
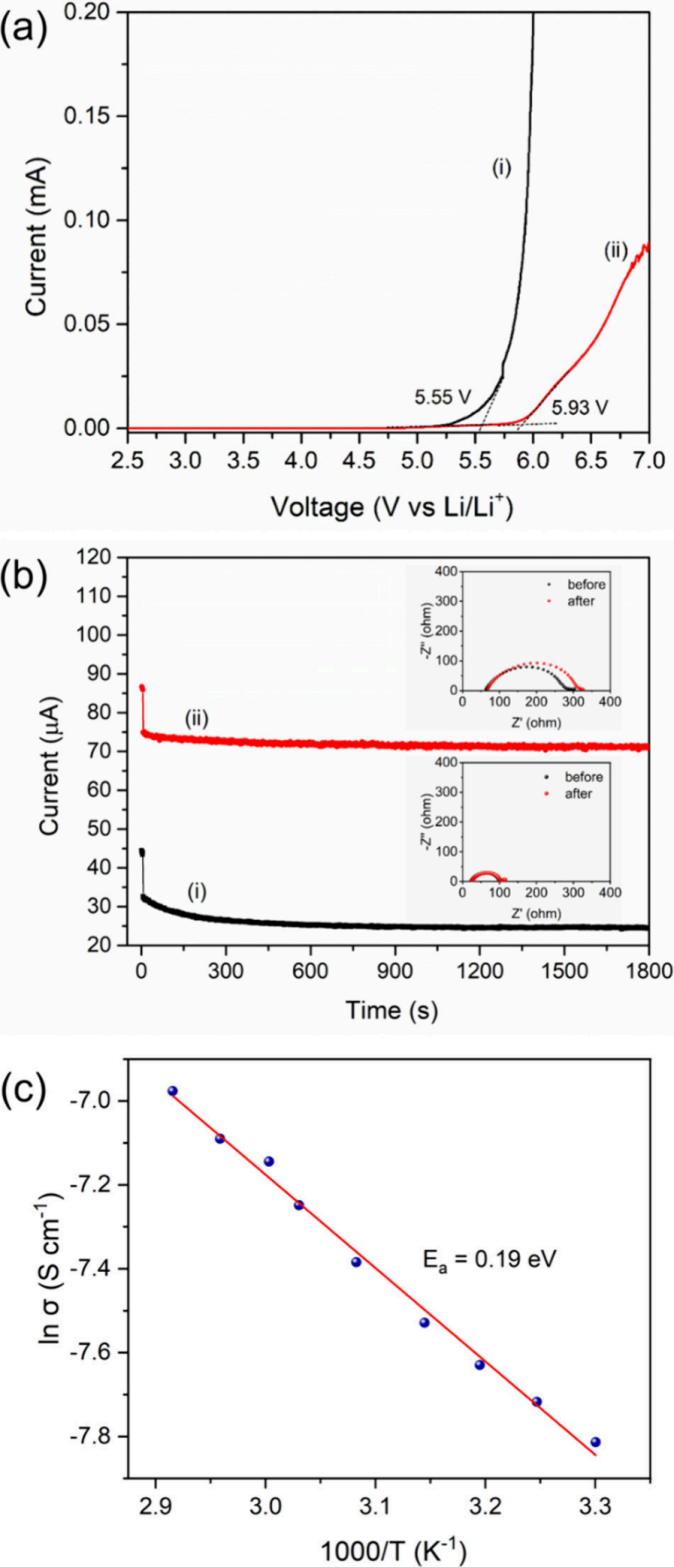
(a) LSV curves of (i) LiTFSI/PVDF-HFP and (ii) LiTFSI/PVDF-HFP/Li-SSP electrolytes. (b) DC polarization profiles of Li||Li cells with (i) LiTFSI/PVDF-HFP and (ii) LiTFSI/PVDF-HFP/Li-SSP electrolytes under 10 mV bias. Insets: Nyquist plots before and after polarization for LiTFSI/PVDF-HFP (top) and LiTFSI/PVDF-HFP/Li-SSP (bottom). (c) Temperature-dependent ionic conductivity of the LiTFSI/PVDF-HFP/Li-SSP electrolyte with Arrhenius fitting (*E*_a_ = 0.19 eV).

In conventional electrolytes, the presence of free anions leads to a low *t*_Li^+^_, resulting in severe concentration polarization. Such polarization not only increases cell resistance but also promotes lithium dendrite growth on the anode.^11,13^ To determine the *t*_Li^+^_ of the Li-SSP composite electrolyte, Li||Li symmetric cells were examined at room temperature using Evans’ method.^13^ The transference number was calculated according to eq 1:

1 $$t_{{\mathrm{Li}}^{+}}=I_{s}\left(\Delta V-I_{0}R_{i0}\right)/I_{0}\left(\Delta V-I_{s}R_{is}\right)$$

where *I*_*0*_ and *I*_*s*_ are the initial and steady-state currents during polarization, respectively, and *R*_*i0*_ and *R*_*is*_ are the interfacial resistances at the initial and steady states, respectively.

Figures 4b­(i) and (ii) display the DC polarization curves of Li||Li cells with the LiTFSI/PVDF-HFP and LiTFSI/PVDF-HFP/Li-SSP electrolytes, respectively, with the corresponding Nyquist plots shown in the insets. Based on eq 1, the *t*_Li^+^_ of the LiTFSI/PVDF-HFP polymer electrolyte is calculated to be 0.18, whereas the Li-SSP composite electrolyte achieves a much higher value of 0.70. This enhancement is attributed to the immobilization of anions within the POP framework. The substantially increased *t*_Li^+^_ indicates that the Li-SSP composite electrolyte can effectively mitigate concentration polarization. Moreover, the SIC-POP matrix may also regulate CEI formation by restricting anion migration, unlike conventional dual-ion electrolytes.

Ionic conductivity (σ) is another crucial factor influencing electrolyte performance. As shown in Figure S17a, the Nyquist plots of the SS|Li-SSP composite electrolyte|SS cell reveal an ionic conductivity of 4.04 × 10^–4^ S cm^–1^ at 30 °C, which is nearly 2 orders of magnitude higher than that of LiTFSI/PVDF-HFP without Li-SSP (4.22 × 10^–6^ S cm^–1^ at 30 °C, Figure S17b). In contrast, the PVDF-HFP/Li-SSP electrolyte without LiTFSI shows a significantly lower conductivity of 3.14 × 10^–6^ S cm^–1^ at 30 °C (Figure S18), likely due to the insufficient lithium-ion concentration in the PVDF-HFP matrix. These results indicate that the enhanced ionic conductivity of the Li-SSP composite electrolyte arises from the synergistic contribution of Li-SSP, LiTFSI, and the PVDF-HFP polymer matrix. The logarithm of ionic conductivity typically varies linearly with the inverse of temperature (1/T), since elevated temperatures provide greater thermal energy for ion migration. To further evaluate lithium-ion transport, ionic conductivity was measured in the range of 30–70 °C. Figure 4c shows a linear Arrhenius relationship between ln σ and 1000/T, yielding an activation energy (*E*_a_) of 0.19 eV. The combination of high ionic conductivity and low ion migration energy confirms that lithium ions in the Li-SSP composite electrolyte can migrate efficiently, enabling rapid and stable ion transport.

### 3.3 Lithium-Ion Diffusion and Interfacial Stability of Li|LFP Cells

Figures 5a and 5b present the first six CV curves of Li|LFP cells with the two electrolytes. In the first cycle, the voltage differences between the oxidation and reduction peaks (ΔV) are 0.44 V for LiTFSI/PVDF-HFP and 0.38 V for LiTFSI/PVDF-HFP/Li-SSP, indicating that the Li-SSP-containing electrolyte offers a smaller ΔV and thus reduced polarization. Moreover, the CV profiles of the LiTFSI/PVDF-HFP cell (Figure 5a) remain unstable even after five cycles, whereas the curves for the Li-SSP-based cell (Figure 5b) stabilize after four cycles. This improvement can be attributed to the single-ion conducting nature of the Li-SSP electrolyte, which lowers interfacial resistance and promotes the formation of robust SEI and CEI films.

**Figure 5:**
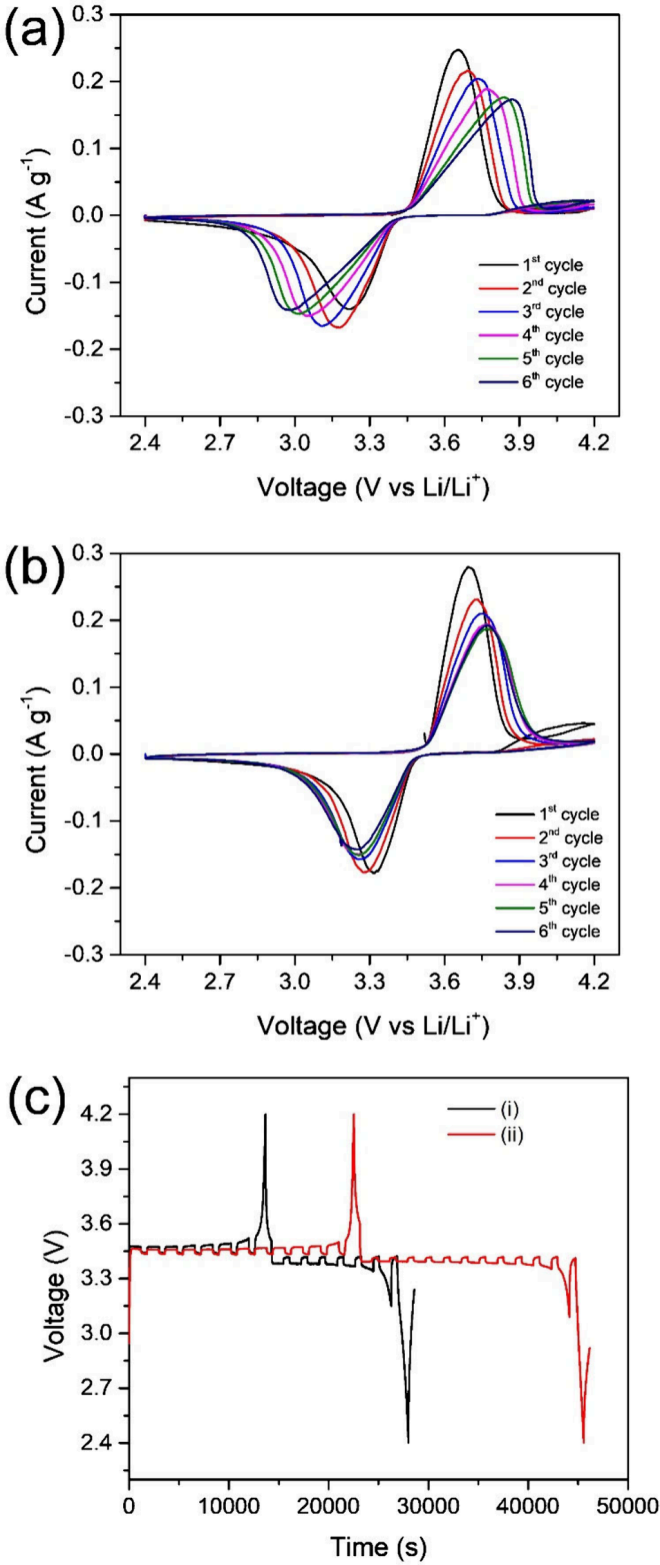
CV curves of Li|LFP cells with (a) LiTFSI/PVDF-HFP and (b) LiTFSI/PVDF-HFP/Li-SSP electrolytes over the first six cycles. (c) GITT profiles of Li|LFP cells with (i) LiTFSI/PVDF-HFP and (ii) LiTFSI/PVDF-HFP/Li-SSP electrolytes.

To further evaluate lithium-ion transport, the diffusion coefficient (*D*_Li^+^_) of Li|LFP cells with the two electrolytes was determined using the GITT. The diffusion coefficient was first calculated according to eq 2:^46,47^

2 $$D_{{Li}^{+}}=\frac{4}{\pi }{\left(\frac{iV_{m}}{z_{A}FS}\right)}^{2}{\left[\frac{\left(dE/d\delta \right)}{\left(dE/d\sqrt{t}\right)}\right]}^{2}$$

where *i* is the applied current (A); *V*_*m*_ is the molar volume of the electrode (cm^3^ mol^–1^); *z*_*A*_ is the charge number; *F* is Faraday’s constant (96485 C mol^–1^); *S* is the electrode/electrolyte contact area (cm^2^); *dE/dδ* is the slope of the coulometric titration curve; and *dE*/*d*√*t* is the slope of the potential–time curve during the current pulse of duration *t*. For cases where *E* (V) varies linearly with √*t*, eq 2 can be simplified to eq 3:

3 $$D_{{Li}^{+}}=\frac{4}{\pi \tau }{\left(\frac{n_{m}V_{m}}{S}\right)}^{2}{\left(\frac{\Delta E_{s}}{\Delta E_{t}}\right)}^{2}$$

where τ is the current pulse duration (s); *n*_*m*_ is the number of moles of active cathode material (mol); Δ*E*_*s*_ is the steady-state voltage change (V); and Δ*E*_*t*_ is the voltage change during the current pulse (V).

As shown in Figure 5c, the GITT measurements were used to calculate *D*_Li^+^_. The obtained values are 1.81 × 10^–12^ cm^2^ s^–1^ for the LiTFSI/PVDF-HFP electrolyte and 5.04 × 10^–12^ cm^2^ s^–1^ for the LiTFSI/PVDF-HFP/Li-SSP electrolyte. The nearly 3-fold enhancement in *D*_Li^+^_ with the Li-SSP-containing electrolyte confirms its superior lithium-ion diffusion capability. These values are consistent with those reported in the literature and underscore the advantage of incorporating a SIC-POP framework to enhance ion mobility, thereby improving the overall electrochemical performance of solid-state batteries.

### 3.4 Rate Capability and Cycle-Life Performance of Li|LFP Cells

Figures 6a and 6b shows the charge–discharge profiles of Li|LFP cells with LiTFSI/PVDF-HFP and LiTFSI/PVDF-HFP/Li-SSP electrolytes at various C-rates. The LiTFSI/PVDF-HFP electrolyte delivers discharge capacities of 143.7, 134.8, and 87.7 mAh g^–1^ at 0.2, 0.5, and 1 C, respectively. However, at higher rates of 2 and 5 C, the capacity drops to nearly zero, reflecting the poor ionic conductivity of the LiTFSI/PVDF-HFP electrolyte. By contrast, the LiTFSI/PVDF-HFP/Li-SSP electrolyte exhibits significantly improved rate performance, achieving 148.1, 142.9, 137.5, 128.8, and 86.7 mAh g^–1^ at 0.2, 0.5, 1, 2, and 5 C, respectively. These results indicate that incorporating Li-SSP into the polymer matrix enhances ionic conductivity and *D*_Li^+^_, thereby enabling more efficient ion transport and better high-rate capability.

**Figure 6:**
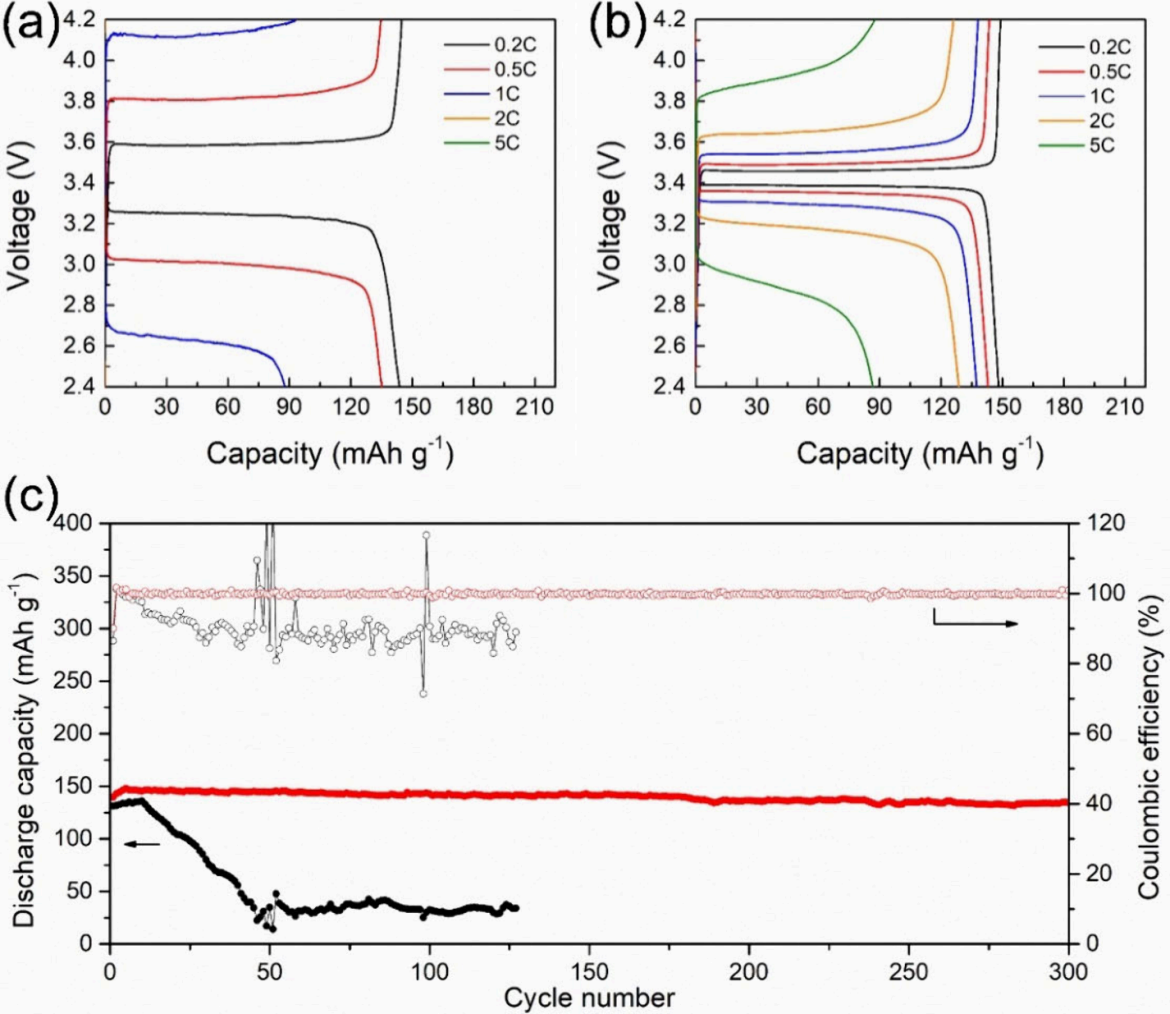
Discharge–charge voltage profiles of (a) Li|LiTFSI/PVDF-HFP|LFP and (b) Li|LiTFSI/PVDF-HFP/Li-SSP|LFP cells at C-rates of 0.2, 0.5, 1, 2, and 5 C. (c) Cycle-life performance of Li|LiTFSI/PVDF-HFP|LFP (black) and Li|LiTFSI/PVDF-HFP/Li-SSP|LFP (red) cells at a charge–discharge rate of 0.5 C at 30 °C.

Figure 6c compares the cycle-life performance of the two electrolytes at 0.5 C and 30 °C. For the LiTFSI/PVDF-HFP electrolyte, the discharge capacity begins to decline sharply after 50 cycles, which can be attributed to limited ionic conductivity and polarization effects inherent to dual-ion conduction. In contrast, the LiTFSI/PVDF-HFP/Li-SSP electrolyte exhibits remarkable stability, retaining ∼ 96.1% of its initial capacity after 300 cycles with nearly 100% Coulombic efficiency. This superior cycling behavior arises from the single-ion conducting nature of Li-SSP, where immobilized anions suppress excessive CEI and SEI growth, reduce interfacial resistance, and facilitate continuous and efficient lithium-ion transport.

### 3.5 Interfacial Resistance and Lithium Plating/Stripping Stability

To further study the enhanced cycle-life performance imparted by Li-SSP, AC impedance analysis was conducted on Li|LFP cells in the fresh state and after 5, 100, and 200 cycles. Figures 7a and 7b present the Nyquist plots of cells with LiTFSI/PVDF-HFP and LiTFSI/PVDF-HFP/Li-SSP electrolytes, respectively. The cell with LiTFSI/PVDF-HFP exhibits severe capacity decay, becoming nearly inactive after 50 cycles; therefore, no impedance data were collected at 200 cycles. In the Nyquist plots, the semicircles correspond to charge-transfer resistance as well as SEI and CEI resistances, while the inclined line at low frequency reflects lithium-ion diffusion. For both systems, the semicircle diameters increase with cycle number, indicating progressive interfacial resistance buildup. However, at the same cycle number, the LiTFSI/PVDF-HFP electrolyte shows much larger semicircles than the Li-SSP-containing system, confirming higher resistances and poorer interfacial stability. To elucidate the differences in interfacial resistance, we evaluated the mechanical properties of the LiTFSI/PVDF-HFP and LiTFSI/PVDF-HFP/Li-SSP electrolytes using nanoindentation to determine their Young’s modulus (Figure S16). The Young’s modulus values of LiTFSI/PVDF-HFP and LiTFSI/PVDF-HFP/Li-SSP are 309 and 217 MPa, respectively, indicating that the Li-SSP-containing electrolyte is mechanically softer, likely due to a reduction in PVDF-HFP crystallinity upon incorporating the porous Li-SSP. A softer electrolyte can better conform to electrode surface asperities, improving interfacial contact and thereby reducing the interfacial resistance.

**Figure 7:**
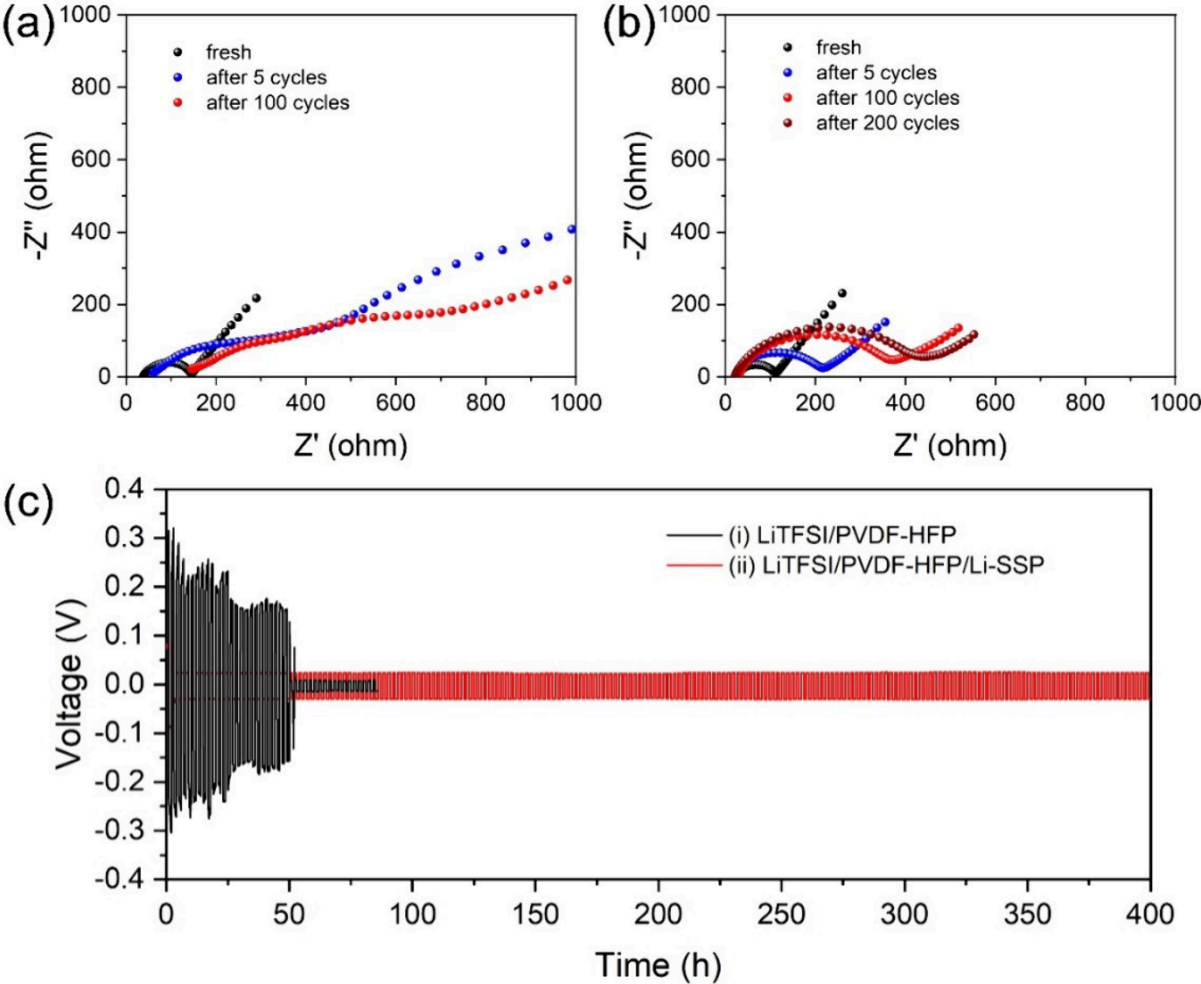
Nyquist plots of (a) Li|LiTFSI/PVDF-HFP|LFP and (b) Li|LiTFSI/PVDF-HFP/Li-SSP|LFP cells in the fresh state and after different cycle numbers. (c) Voltage profiles of symmetric Li||Li cells during plating/stripping with (i) LiTFSI/PVDF-HFP and (ii) LiTFSI/PVDF-HFP/Li-SSP electrolytes.

The stability of the lithium electrode was further examined in Li||Li symmetric cells at a plating/stripping capacity of 0.5 mAh cm^–2^ (Figure 7c).^48^ In the LiTFSI/PVDF-HFP system, the overpotential gradually increases with cycling, followed by a sudden voltage drop after ∼ 50 h, leading to rapid cell failure. In contrast, the LiTFSI/PVDF-HFP/Li-SSP cell maintains a stable and low overpotential throughout continuous cycling, operating for up to 400 h without short-circuiting. This remarkable stability is attributed to the single-ion conducting nature of Li-SSP, where the immobilized anionic framework reduces concentration polarization, suppresses parasitic anion reduction, and inhibits the formation of resistive SEI layers. Consequently, the Li-SSP composite electrolyte ensures prolonged and stable lithium plating/stripping behavior, in line with its superior cycling performance observed in the Li|LFP cell performance.

To evaluate the effectiveness of the composite electrolyte in suppressing lithium dendrite growth, critical current density (CCD) measurements were conducted using symmetric Li|Li cells with either LiTFSI/PVDF-HFP or LiTFSI/PVDF-HFP/Li-SSP as the electrolyte. The voltage–time profiles recorded under stepwise increases in current density are presented in Figure S20. For the LiTFSI/PVDF-HFP electrolyte, stable lithium plating/stripping behavior was maintained only up to 0.2 mA cm^–2^. At higher current densities, a sudden voltage drop was observed, indicating internal short-circuiting caused by lithium dendrite penetration. This relatively low CCD can be attributed to limited ion-transport regulation within the polymer matrix and suboptimal interfacial contact between the electrolyte and lithium electrodes. In contrast, the LiTFSI/PVDF-HFP/Li-SSP composite electrolyte exhibited markedly enhanced stability, sustaining reversible lithium plating/stripping up to 0.5 mA cm^–2^ before short-circuit failure. The improved CCD is ascribed to the incorporation of Li-SSP, which enhances Li^+^ transport and promotes more homogeneous interfacial contact, thereby reducing local current density fluctuations. These factors collectively delay dendrite nucleation and propagation, extending the operational current limit of the symmetric cell. Overall, the CCD results clearly demonstrate that the incorporation of Li-SSP substantially enhances the dendrite-suppression capability of the electrolyte, supporting its role as an effective ionically favorable component in solid-state lithium battery electrolytes.

To gain further insight into the resistance components of the cells, DRT analysis was employed to determine appropriate equivalent circuit models for EIS fitting, thereby providing a more detailed explanation of the impedance results. For the Li|LiTFSI/PVDF-HFP|LFP cell prior to cycling, the DRT plot (Figure 8a) displays three main peaks, which can be assigned to SEI resistance, charge-transfer resistance, and lithium-ion diffusion. After 5 and 100 cycles (Figures 8b and 8c), the peaks become more pronounced, indicating increased resistance, and up to seven peaks are observed in each DRT spectrum. According to previous report employing three-electrode configurations, up to seven distinct peaks can typically be resolved in the DRT analysis of batteries.^49^ In this work, nearly seven major peaks are consistently observed. The peak around τ ∼ 10^–5^ s may be associated with contact resistance between electrode particles. Peaks at τ_3_ and τ_4_ correspond to SEI and CEI resistances, respectively, while peaks at τ_5_ and τ_6_ are assigned to charge-transfer processes at the cathode and anode. The dominant peak at τ ≥ 0.1 s is attributed to lithium-ion diffusion, whereas other peaks are linked to polarization resistances corresponding to the semicircles in the Nyquist plots.^50,51^ Thus, the stronger DRT features observed in Figures 8b and 8c suggest that for the LiTFSI/PVDF-HFP electrolyte, prolonged cycling leads to increased SEI/CEI growth and hindered lithium diffusion. In comparison, the DRT plot of the Li|LiTFSI/PVDF-HFP/Li-SSP|LFP cell before cycling (Figure 8d) resembles that of the LiTFSI/PVDF-HFP system. However, after extended cycling (Figures 8e and 8f), the DRT peaks become weaker, indicating reduced resistance buildup. This result demonstrates that the incorporation of Li-SSP mitigates interfacial and charge-transfer resistances. Based on the evolution of the peaks, we infer that τ_3_ is primarily associated with CEI film formation at the LFP electrode during cycling, consistent with reports in the literature.^52,53^

**Figure 8:**
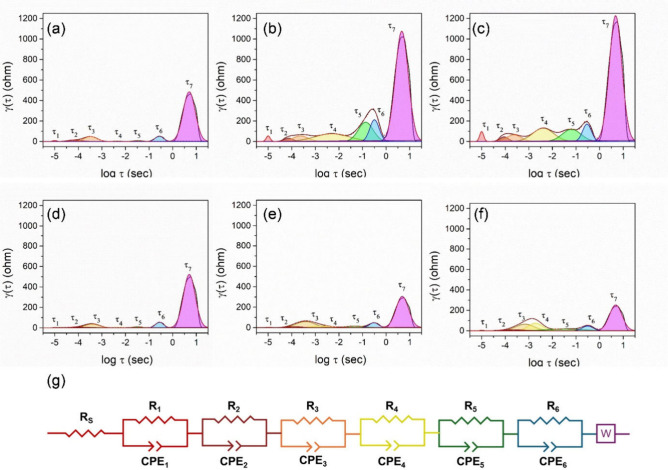
DRT plots corresponding to the EIS results of (a–c) Li|LiTFSI/PVDF-HFP|LFP and (d–f) Li|LiTFSI/PVDF-HFP/Li-SSP|LFP cells in the fresh state and after the fifth, and 100th cycles. (g) Equivalent circuit model for the Li|LFP cells.

According to the DRT results, an equivalent circuit model for Li|LFP cells with different electrolytes was established, as shown in Figure 8g. The fitted resistance values are summarized in Table 1. R_s_ and R_1_ correspond to τ_1_, while R_2_, R_3_, and R_4_ are associated with τ_2_, τ_3_, and τ_4_, respectively. R_total_ represents the overall resistance of the cell. In the fresh state, the R_total_ values of the LiTFSI/PVDF-HFP and LiTFSI/PVDF-HFP/Li-SSP cells are 147.0 and 119.5 Ω, respectively. With increasing cycle number, the R_total_ of the LiTFSI/PVDF-HFP cell rises sharply, primarily due to the growth of R_3_ and R_4_, which are attributed to CEI film resistance and charge-transfer resistance, respectively. In contrast, the LiTFSI/PVDF-HFP/Li-SSP cell shows only a gradual increase in R_total_. These results demonstrate that the incorporation of Li-SSP effectively suppresses resistance growth, particularly at the cathode interface, by reducing CEI formation and charge-transfer impedance, thereby lowering overall cell resistance and enhancing electrochemical performance.

**Table 1:** Resistance Values (R_s_, R_1_, R_2_, R_3_, R_4_, R_5_, and R_6_) Simulated from Nyquist Plots for Li|LFP Cells with Different Electrolytes in the Fresh State and after the 5th and 100th Cycles

		Resistance (ohm)							
Cycle	Electrolyte	R_S_	R_1_	R_2_	R_3_	R_4_	R_5_	R_6_	R_total_
Fresh	LiTFSI/PVDF-HFP	10.7	9.1	20.4	63.5	3.4	8.6	44.5	160.2
	LiTFSI/PVDF-HFP/Li-SSP	7.6	7.4	17.5	62.0	3.9	10.7	50.7	159.8
5th	LiTFSI/PVDF-HFP	21.2	17.2	36.1	79.7	130.4	145.7	61.0	491.2
	LiTFSI/PVDF-HFP/Li-SSP	10.3	10.2	15.1	125.8	37.2	29.6	48.5	276.6
100th	LiTFSI/PVDF-HFP	37.3	29.2	65.3	149.9	460.9	374.5	256.1	1373.1
	LiTFSI/PVDF-HFP/Li-SSP	13.27	13.8	22.5	125.5	111.1	65.8	53.8	405.7

The EIS and DRT results indicate that the resistance associated with the CEI on the LFP electrode is lower when using the Li-SSP electrolyte. To further confirm CEI formation on the cathode, morphology characterization of the LFP electrode was conducted by SEM and TEM. Figures 9a–9c show SEM images of the pristine LFP electrode, the electrode after 200 cycles in the LiTFSI/PVDF-HFP electrolyte, and the electrode after 200 cycles in the LiTFSI/PVDF-HFP/Li-SSP electrolyte, respectively. Before cycling, the surface of the LFP particles is smooth (Figure 9a). After 200 cycles in the LiTFSI/PVDF-HFP electrolyte, the LFP surface becomes rough, indicative of a thick CEI layer. In contrast, the surface of the LFP cycled in the Li-SSP electrolyte appears smoother, suggesting that the SIC-POP framework suppresses excessive CEI formation by immobilizing anions in the single-ion conductor. TEM images (Figures 9d–9f) further support these observations. After 200 cycles in the LiTFSI/PVDF-HFP electrolyte, a thick CEI film (∼5.79 nm) forms on the LFP surface. However, in the LiTFSI/PVDF-HFP/Li-SSP system, the CEI thickness is significantly reduced (∼2.72 nm). Together, the SEM and TEM analyses confirm that the Li-SSP electrolyte limits CEI growth, consistent with the lower resistances observed in the DRT and EIS results.

**Figure 9:**
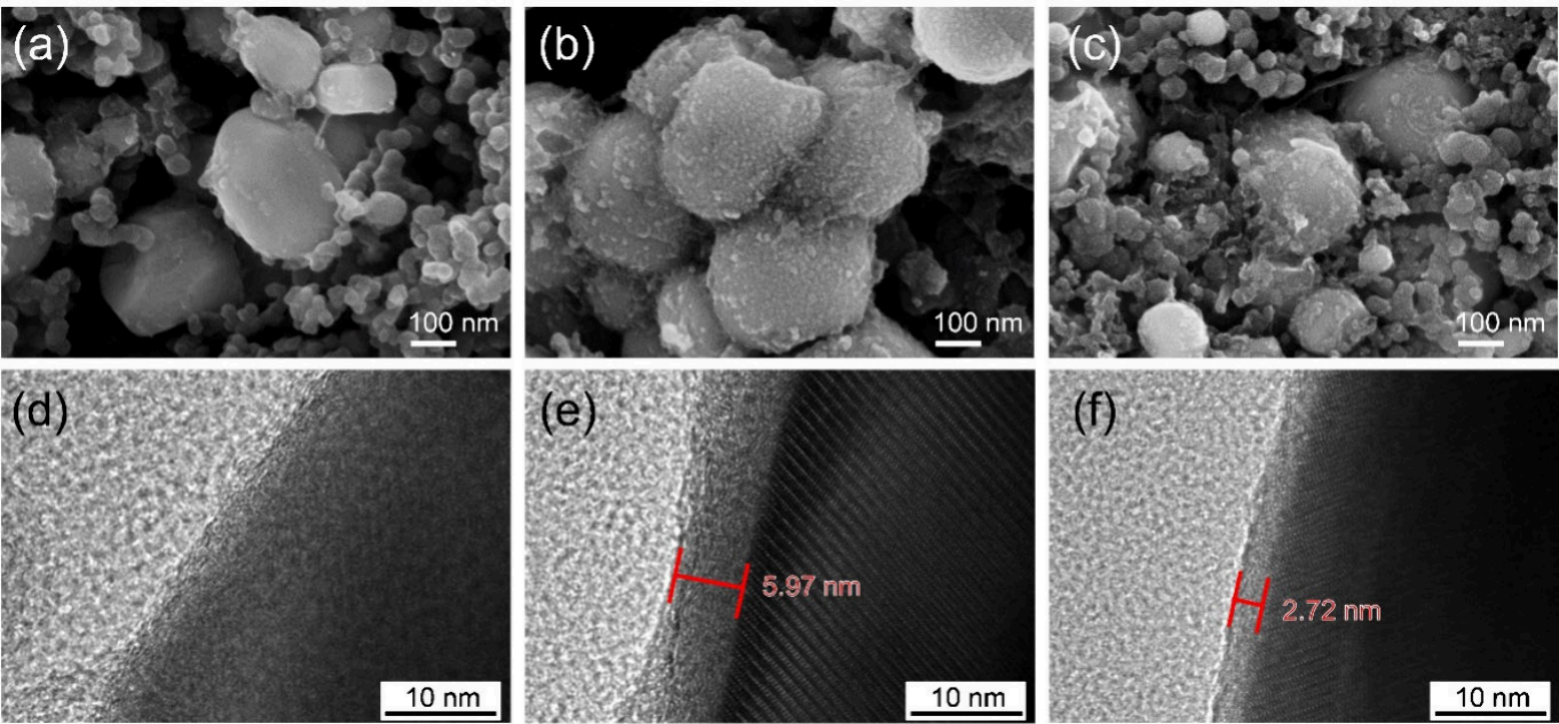
SEM images of LFP electrodes: (a) pristine, (b) after 200 cycles with LiTFSI/PVDF-HFP, and (c) after 200 cycles with LiTFSI/PVDF-HFP/Li-SSP electrolytes. TEM images of LFP electrodes: (d) pristine, (e) after 200 cycles with LiTFSI/PVDF-HFP, and (f) after 200 cycles with LiTFSI/PVDF-HFP/Li-SSP electrolytes.

To investigate the chemical composition of the CEI films, the LFP electrodes were analyzed by XPS. As shown in the survey spectra (Figures S22–S24), the pristine electrode shows a weak O 1s peak and a strong C 1s peak. After 200 cycles, however, the LFP electrodes cycled in LiTFSI/PVDF-HFP and LiTFSI/PVDF-HFP/Li-SSP electrolytes exhibit markedly different spectra, reflecting the formation of CEI layers on the electrode surface. High-resolution XPS spectra were collected to further examine the chemical states of the CEI films. For the pristine electrode, the C 1s spectrum (Figure 10a) shows three peaks at 284.8, 285.8, and 290.6 eV, corresponding to CH_*x*_/C–C, C–O, and C–F species, respectively. The O 1s spectrum (Figure 10b) presents two peaks at 531.8 and 533.7 eV, assigned to P–O and C–O bonds. The F 1s spectrum (Figure 10c) exhibits a single peak at 687.8 eV, attributed to the C–F bonds of PVDF. After 200 cycles in the LiTFSI/PVDF-HFP electrolyte, additional species appear (Figures 10d–10f). The C 1s spectrum shows five peaks at 284.8, 285.9, 289.0, 290.7, and 291.7 eV, corresponding to CH_*x*_/C–C, C–O, C=O, C–F, and −CF_3_ groups, respectively. The O 1s spectrum displays peaks at 530.1, 531.8, and 533.8 eV, assigned to Li_2_O, P–O/C=O, and C–O species. In the F 1s spectrum, three peaks at 685.6, 688.0, and 688.9 eV are identified as LiF, C–F, and – CF_3_, respectively. These results indicate that the CEI film largely originates from the decomposition of LiTFSI and a minor fraction of carbonate solvent (EC/PC), which contributes to interfacial passivation. For the LFP electrode cycled in the LiTFSI/PVDF-HFP/Li-SSP electrolyte (Figures 10g–10i), the overall peak patterns are similar to those observed in the LiTFSI/PVDF-HFP system, suggesting comparable CEI chemical composition. However, the survey spectrum (Figure S24) reveals a stronger PVDF-derived F peak, implying that the CEI film is relatively thinner when Li-SSP is present. A thinner CEI layer reduces interfacial resistance, which correlates with the improved electrochemical performance of the Li-SSP-containing system. Therefore, the XPS results confirm that CEI formation in both electrolytes arises primarily from the decomposition of LiTFSI and carbonate solvents. The incorporation of Li-SSP suppresses excessive CEI buildup, yielding a thinner interphase that lowers resistance and enhances cycling stability and overall cell performance.

**Figure 10:**
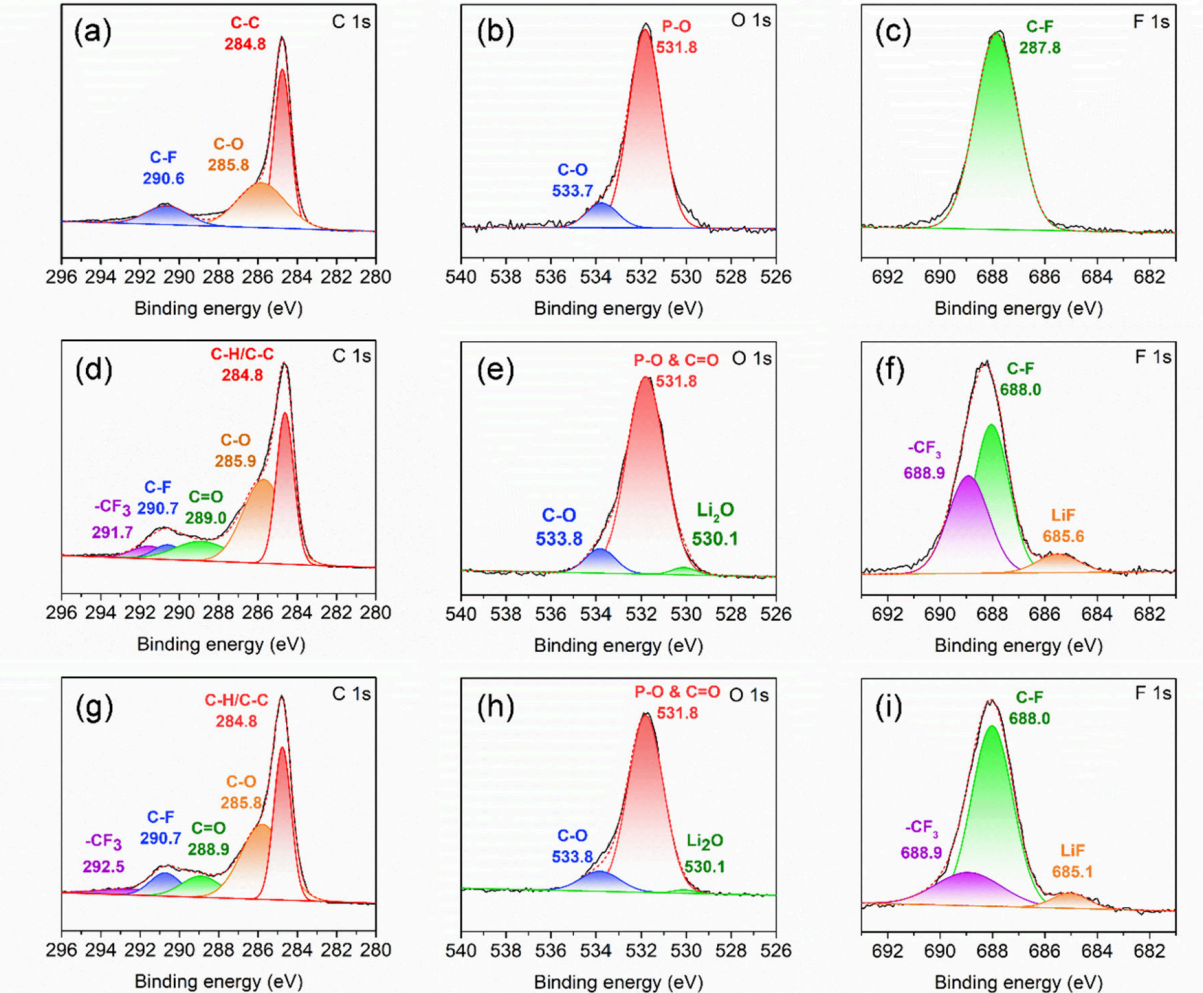
High-resolution XPS spectra of (a–c) C 1s, F 1s, and O 1s for the pristine LFP electrode; (d–f) corresponding spectra for the LFP electrode after 200 cycles in the LiTFSI/PVDF-HFP electrolyte; and (g–i) corresponding spectra for the LFP electrode after 200 cycles in the LiTFSI/PVDF-HFP/Li-SSP electrolyte.

## 4 Conclusion

In this study, a SIC-POP was successfully synthesized via a Sonogashira coupling reaction between Li-BPSSA linkers and a triethynylbenzene core, forming a three-dimensional porous network. The structure of SIC-POP was confirmed through FT-IR and BET analyses. When incorporated into a LiTFSI/PVDF-HFP solid electrolyte, the resulting LiTFSI/PVDF-HFP/SIC-POP composite exhibited significantly enhanced ionic conductivity and a higher lithium-ion transference number. Electrochemical evaluation demonstrated that cells with the SIC-POP-containing electrolyte delivered improved C-rate capability and superior cycling stability. Symmetric Li||Li cell tests further revealed that the composite electrolyte effectively stabilized the lithium electrode interface during plating/stripping. Moreover, EIS and DRT analyses indicated suppressed SEI and charge-transfer resistances, which were consistent with SEM and TEM observations of thinner and more uniform interfacial layers. Overall, these results highlight the promise of SIC-POP as a functional additive for advancing the performance, interfacial stability, and durability of next-generation solid polymer electrolytes in high-performance lithium-ion batteries.

## Supplementary Material



## Notes

^1^H, ^7^Li, and ^13^C NMR spectra of intermediates; HRMS spectra of intermediates; TGA of Li-SSP; photographs of the solid-state electrolytes; Nyquist plots of the solid-state electrolytes; CCD of Li|solid-state electrolyte|Li cells; nanoindentation measurements of solid-state electrolytes; GITT profiles of cells; cycle profiles of cells; XPS survey spectra of LFP electrodes (PDF)

## References

[1] Chen Y., Kang Y., Zhao Y., Wang L., Liu J., Li Y., Liang Z., He X., Li X., Tavajohi N., Li B. (2021). A review of lithium-ion battery safety concerns: The issues, strategies,
and testing standards. J. Energy Chem..

[2] Doughty D. H., Roth E. P. (2012). A General Discussion
of Li Ion Battery Safety. Electrochem. Soc.
Interface.

[3] Liu K., Liu Y., Lin D., Pei A., Cui Y. (2018). Materials for lithium-ion
battery safety. Sci. Adv..

[4] Fan L.-Z., He H., Nan C.-W. (2021). Tailoring
inorganic–polymer composites for the
mass production of solid-state batteries. Nat.
Rev. Mater..

[5] Bates A. M., Preger Y., Torres-Castro L., Harrison K. L., Harris S. J., Hewson J. (2022). Are solid-state batteries safer than lithium-ion batteries?. Joule.

[6] Li C.-C. (2025). Dispersants
and particle dispersion uniformity in lithium batteries: from slurry
formulation to solid-state design. Energy Storage
Mater..

[7] Xu L., Tang S., Cheng Y., Wang K., Liang J., Liu C., Cao Y.-C., Wei F., Mai L. (2018). Interfaces in Solid-State
Lithium Batteries. Joule.

[8] Zhou D., Shanmukaraj D., Tkacheva A., Armand M., Wang G. (2019). Polymer Electrolytes
for Lithium-Based Batteries: Advances and Prospects. Chem..

[9] Ding Y., He B., Wang D., Avdeev M., Li Y., Shi S. (2023). Software for
Evaluating Ionic Conductivity of Inorganic–Polymer Composite
Solid Electrolytes. Energy Mater. Adv..

[10] Wang S., La Monaca A., Demopoulos G. P. (2025). Composite solid-state electrolytes
for all solid state lithium batteries: progress, challenges and outlook. Energy Adv..

[11] Ye H., Huang J., Xu J. J., Khalfan A., Greenbaum S. G. (2007). Li Ion
Conducting Polymer Gel Electrolytes Based on Ionic Liquid/PVDF-HFP
Blends. J. Electrochem. Soc..

[12] Fergus J. W. (2010). Ceramic
and polymeric solid electrolytes for lithium-ion batteries. J. Power Sources.

[13] Quartarone E., Mustarelli P. (2011). Electrolytes
for solid-state lithium rechargeable batteries:
recent advances and perspectives. Chem. Soc.
Rev..

[14] Ma Y., Wu F., Chen N., Ma Y., Yang C., Shang Y., Liu H., Li L., Chen R. (2022). Reversing the dendrite growth direction
and eliminating the concentration polarization via an internal electric
field for stable lithium metal anodes. Chem.
Sci..

[15] Gao J., Wang C., Han D.-W., Shin D.-M. (2021). Single-ion conducting
polymer electrolytes as a key jigsaw piece for next-generation battery
applications. Chem. Sci..

[16] Deng K., Zeng Q., Wang D., Liu Z., Qiu Z., Zhang Y., Xiao M., Meng Y. (2020). Single-ion
conducting
gel polymer electrolytes: design, preparation and application. J. Mater. Chem. A.

[17] Zhu J., Zhang Z., Zhao S., Westover A. S., Belharouak I., Cao P.-F. (2021). Single-Ion Conducting Polymer Electrolytes for Solid-State
Lithium–Metal Batteries: Design, Performance, and Challenges. Adv. Energy Mater..

[18] Rohan R., Kuo T.-C., Chen M.-W., Lee J.-T. (2017). Nanofiber Single-Ion
Conducting Electrolytes: An Approach for High-Performance Lithium
Batteries at Ambient Temperature. ChemElectroChem..

[19] Sun Z., Xi K., Chen J., Abdelkader A., Li M.-Y., Qin Y., Lin Y., Jiang Q., Su Y.-Q., Vasant Kumar R., Ding S. (2022). Expanding the active charge carriers of polymer electrolytes in lithium-based
batteries using an anion-hosting cathode. Nat.
Commun..

[20] He Y., Wang C., Zou P., Lin R., Hu E., Xin H. L. (2023). Anion-tethered Single Lithium-ion
Conducting Polyelectrolytes
through UV-induced Free Radical Polymerization for Improved Morphological
Stability of Lithium Metal Anodes. Angew. Chem.,
Int. Ed..

[21] Song J., Lee H., Choo M.-J., Park J.-K., Kim H.-T. (2015). Ionomer-Liquid Electrolyte
Hybrid Ionic Conductor for High Cycling Stability of Lithium Metal
Electrodes. Sci. Rep..

[22] Liao B.-C., Jian B.-H., Wu M.-J., Lee J.-T. (2023). Designing 3D Porous
Organic Polymers for High-Performance Organic Battery Cathodes. ACS Appl. Energy Mater..

[23] Mohamed M. G., Chaganti S. V., Li M.-S., Samy M. M., Sharma S. U., Lee J.-T., Elsayed M. H., Chou H.-H., Kuo S.-W. (2022). Ultrastable
Porous Organic Polymers Containing Thianthrene and Pyrene Units as
Organic Electrode Materials for Supercapacitors. ACS Appl. Energy Mater..

[24] Mohamed M. G., EL-Mahdy A. F. M., Kotp M. G., Kuo S.-W. (2022). Advances in porous
organic polymers: syntheses, structures, and diverse applications. Mater. Adv..

[25] Chen L., Ding K., Li K., Li Z., Zhang X., Zheng Q., Cai Y.-P., Lan Y.-Q. (2022). Crystalline Porous
Materials-based Solid-State Electrolytes for Lithium Metal Batteries. EnergyChem..

[26] DeBlase C. R., Silberstein K. E., Truong T.-T., Abruna H. D., Dichtel W. R. (2013). β-Ketoenamine-Linked
Covalent Organic Frameworks Capable of Pseudocapacitive Energy Storage. J. Am. Chem. Soc..

[27] Luo Z., Liu L., Ning J., Lei K., Lu Y., Li F., Chen J. (2018). A Microporous Covalent–Organic Framework with Abundant Accessible
Carbonyl Groups for Lithium-Ion Batteries. Angew.Chem.
Int. Ed..

[28] Qin W.-M., Li Z., Su W.-X., Hu J.-M., Zou H., Wu Z., Ruan Z., Cai Y.-P., Li K., Zheng Q. (2025). Porous Organic
Cage-Based Quasi-Solid-State Electrolyte with Cavity-Induced Anion-Trapping
Effect for Long-Life Lithium Metal Batteries. Nano-Micro Lett..

[29] Li Z., Wang L., Liu Y., Yu M., Liu B., Men Y., Sun Z., Hu W., Zhu G. (2023). Single-Ion Polymer
Electrolyte Based on Lithium-Rich Imidazole Anionic Porous Aromatic
Framework for High Performance Lithium-Ion Batteries. Small.

[30] Li Z., Wang L., Yu M., Liu Y., Liu B., Sun Z., Hu W., Zhu G. (2022). Lithium-Rich Porous Aromatic Framework-Based
Quasi-Solid Polymer Electrolyte for High-Performance Lithium Ion Batteries. ACS Appl. Mater. Interfaces.

[31] Li S., Wang L., Liu C., Liu Y., Li Z., Liu B., Sun Z., Hu W. (2024). Lithium-Rich
Porous Aromatic Framework
Doped Quasi-Solid Polymer Electrolyte for Lithium Battery with High
Cycling Stability. ACS Appl. Mater. Interfaces.

[32] Liu C., Li Z., Liu Y., Liu B., Ni C., Sun Z., Hu W., Chen H., Zhu G. (2025). Amino-containing lithium-rich PAF
doped single-ion polymer electrolytes for improved lithium dendrite
inhibition and electrochemical performance of lithium batteries. J. Membr. Sci..

[33] Saleem A., Iqbal R., Majeed M. K., Hussain A., Akbar A. R., Hussain Z., Jabar B., Rauf S., Shaw L. L. (2024). Boosting
lithium-ion conductivity of polymer electrolyte by selective introduction
of covalent organic frameworks for safe lithium metal batteries. Nano Energy.

[34] Yu M., Liu Y., Wang L., Cui F., Liu B., Hu W., Lu Y., Zhu G. (2025). Porphyrin-framed PAF Based Single-Ion
Lithium Salt
Boosting Quasi Solid-State Lithium-Ion Battery Performance at Low
Temperatures. Adv. Energy Mater..

[35] Du Y., Yang H., Whiteley J. M., Wan S., Jin Y., Lee S.-H., Zhang W. (2016). Ionic Covalent Organic
Frameworks
with Spiroborate Linkage. Angew. Chem., Int.
Ed..

[36] Lee J.-H., Lee H., Lee J., Kang T. W., Park J. H., Shin J.-H., Lee H., Majhi D., Lee S. U., Kim J.-H. (2023). Multicomponent Covalent
Organic Framework Solid Electrolyte Allowing Effective Li-Ion Dissociation
and Diffusion for All-Solid-State Batteries. ACS Nano.

[37] Liu C., Fang X., Peng H., Huang W., Liu W., Yang Y., Li Y. (2025). A single-ion conductive composite
gel electrolyte based on helical mesoporous silica nanofibers for
high-performance lithium-ion batteries. J. Colloid
Interface Sci..

[38] Porcarelli L., Sutton P., Bocharova V., Aguirresarobe R. H., Zhu H., Goujon N., Leiza J. R., Sokolov A., Forsyth M., Mecerreyes D. (2021). Single-Ion
Conducting Polymer Nanoparticles as Functional
Fillers for Solid Electrolytes in Lithium Metal Batteries. ACS Appl. Mater. Interfaces.

[39] Kross R. D., Fassel V. A., Margoshes M. (1956). The Infrared
Spectra of Aromatic
Compounds. II. Evidence Concerning the Interaction of π-Electrons
and σ-Bond Orbitals in C-H Out-of-plane Bending Vibrations. J. Am. Chem. Soc..

[40] Mooney E. F. (1964). The infrared
spectra of chlorobeneene and bromobenzene derivatives-III. Toluenes. Spectrochim. Acta.

[41] Ahmed F., Choi I., Ryu T., Yoon S., Rahman M. M., Zhang W., Jang H., Kim W. (2020). Highly conductive
divalent
fluorosulfonyl imide based electrolytes improving Li-ion battery performance:
Additive potentiating electrolytes action. J.
Power Sources.

[42] Gharehkhani A., Ghorbani-vaghei R., Alavinia S. (2021). Synthesis of calixresorcarenes using
magnetic polytriazine-benzene sulfonamide-SO_3_H. RSC Adv..

[43] Markiewicz R., Klimaszyk A., Jarek M., Taube M., Florczak P., Kempka M., Fojud Z., Jurga S. (2021). Influence of Alkyl
Chain Length on Thermal Properties, Structure, and Self-Diffusion
Coefficients of Alkyltriethylammonium-Based Ionic Liquids. Int. J. Mol. Sci..

[44] Laybourn A., Dawson R., Clowes R., Hasell T., Cooper A. I., Khimyak Y. Z., Adams D. J. (2014). Network formation mechanisms in conjugated
microporous polymers. Polym. Chem..

[45] Lingua G., Shevtsov V. Y., Vlasov P. S., Puchot L., Gerbaldi C., Shaplov A. S. (2024). A New (Trifluoromethane)­Sulfonylimide
Single-Ion Conductor
with PEG Spacer for All-Solid-State Lithium-Based Batteries. ACS Mater. Lett..

[46] Zheng J., Shi W., Gu M., Xiao J., Zuo P., Wang C., Zhang J.-G. (2013). Electrochemical
Kinetics and Performance of Layered
Composite Cathode Material Li­[Li_0.2_Ni_0.2_Mn_0.6_]­O_2_. J. Electrochem. Soc..

[47] Chen Y., Wang L., Anwar T., Zhao Y., Piao N., He X., Zhu Q. (2017). Application of Galvanostatic Intermittent Titration
Technique to Investigate Phase Transformation of LiFePO_4_ Nanoparticles. Electrochim. Acta.

[48] Pan C.-Y., Kuo G.-L., Li C.-C. (2025). Solid Electrolytes
and Dendrite Dynamics
in Solid-State Lithium–Sulfur Batteries. ACS Appl.Mater. Interfaces.

[49] Wu Y.-W., Li C.-C. (2025). Electrochemical
assessment of a Li-ion full cell with cathode-anode
impedance separation via in-situ EIS-DRT and three-electrode configuration. J. Power Sources.

[50] Hahn M., Rosenbach D., Krimalowski A., Nazarenus T., Moos R., Thelakkat M., Danzer M. A. (2020). Investigating solid
polymer and ceramic electrolytes for lithium-ion batteries by means
of an extended Distribution of Relaxation Times analysis. Electrochim. Acta.

[51] Kumchompoo J., Lee J.-T., Li C.-C. (2024). How dispersed
LLZTO enhances ionic
conductivity in LiFePO_4_ composite cathodes for solid-state
batteries. J. Energy Storage.

[52] Wang X., Chen C., Wu S., Zheng H., Chen Y., Liu H., Wu Y., Duan H. (2022). High-Rate and Long-Life Au Nanorods/LiFePO_4_ Composite
Cathode for Lithium-Ion Batteries. Energy Technol..

[53] Iurilli P., Brivio C., Wood V. (2022). Detection
of Lithium-Ion Cells’
Degradation through Deconvolution of Electrochemical Impedance Spectroscopy
with Distribution of Relaxation Time. Energy
Technol..

