# Supplementary material for: Sulfonylimide-Based Single-Ion-Conducting Porous Organic Polymer Electrolytes for Enhanced Performance of Solid-State Lithium Batteries
Source: ACS Appl Mater Interfaces. 2026 Feb 10;18(7):11259–72. doi: 10.1021/acsami.5c20914 (PMC12954658; doi:10.1021/acsami.5c20914)
Supplement: Supplementary file 1 [file am5c20914_si_001.pdf]

Supporting Information

Sulfonylimide-Based Single-Ion-Conducting Porous Organic Polymer Electrolytes for Enhanced Performance of Solid-State Lithium Batteries

Pin-Jyun Chen,[a] Jaturon Kumchompoo[b], Bo-Lin Chen,[a] Yun-Chen Chuang,[a] Bei-Chun Liao,[a] Chia-Chen Li,[c] Jyh-Tsung Lee[a,d]*

[a]Department of Chemistry, National Sun Yat-sen University, Kaohsiung 80424, Taiwan

[b]Department of Chemistry, Faculty of Science and Technology, Thammasat University, Pathumtani 12120, Thailand

[c]Department of Materials Science and Engineering, National Tsing Hua University, Hsinchu 300044, Taiwan

[d]Department of Medicinal and Applied Chemistry, Kaohsiung Medical University, Kaohsiung 80708, Taiwan

**Corresponding author*

E-mail: jtlee@faculty.nsysu.edu.tw

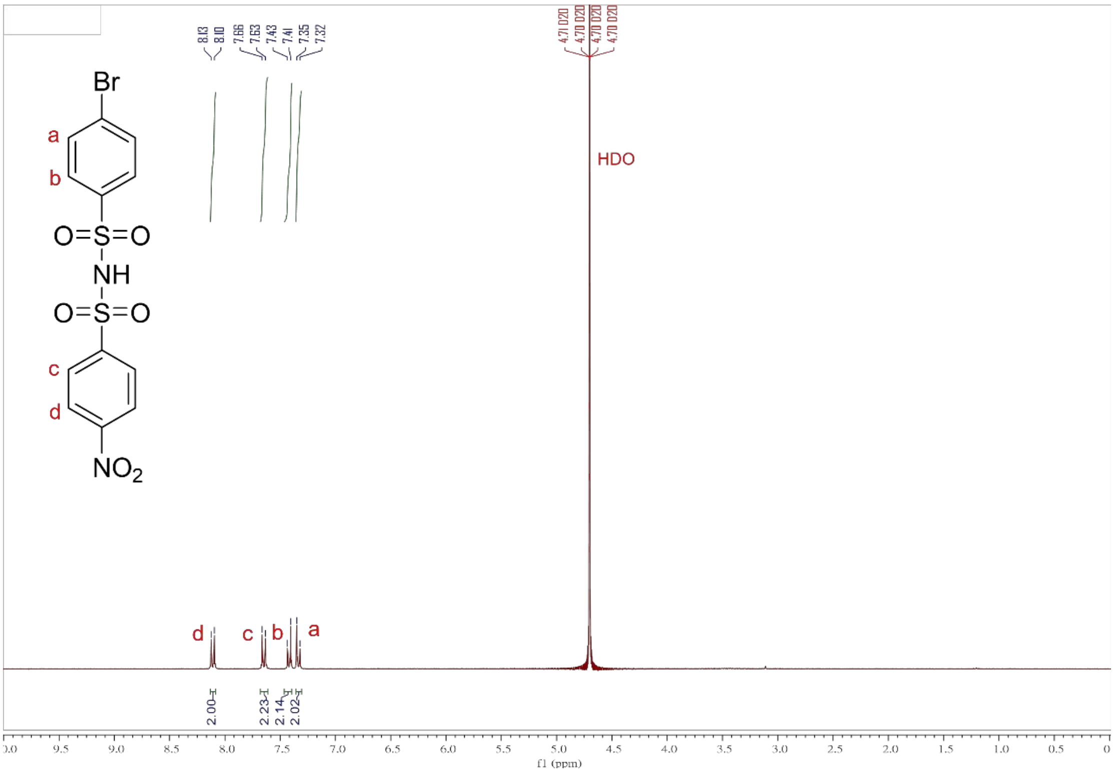

Figure S1. ^{1}H NMR spectrum of BNBSA.

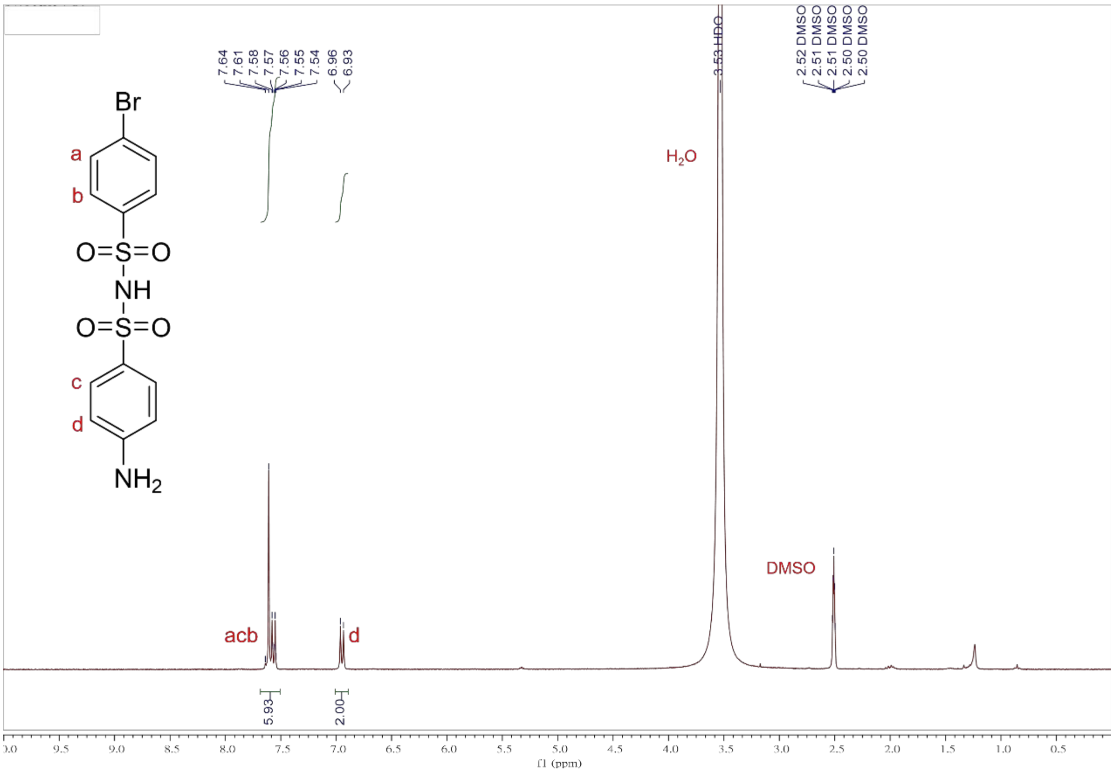

Figure S2. ^{1}H NMR spectrum of BABSA.

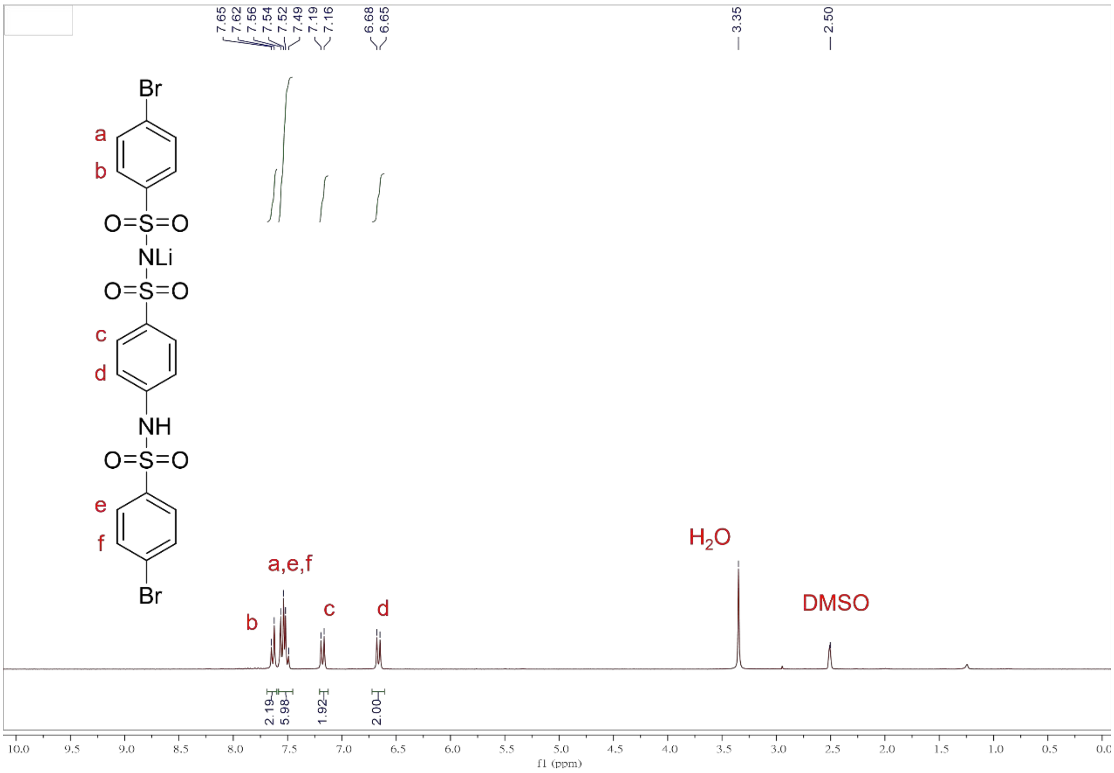

Figure S3. ^{1}H NMR spectrum of Li-BPSSA.

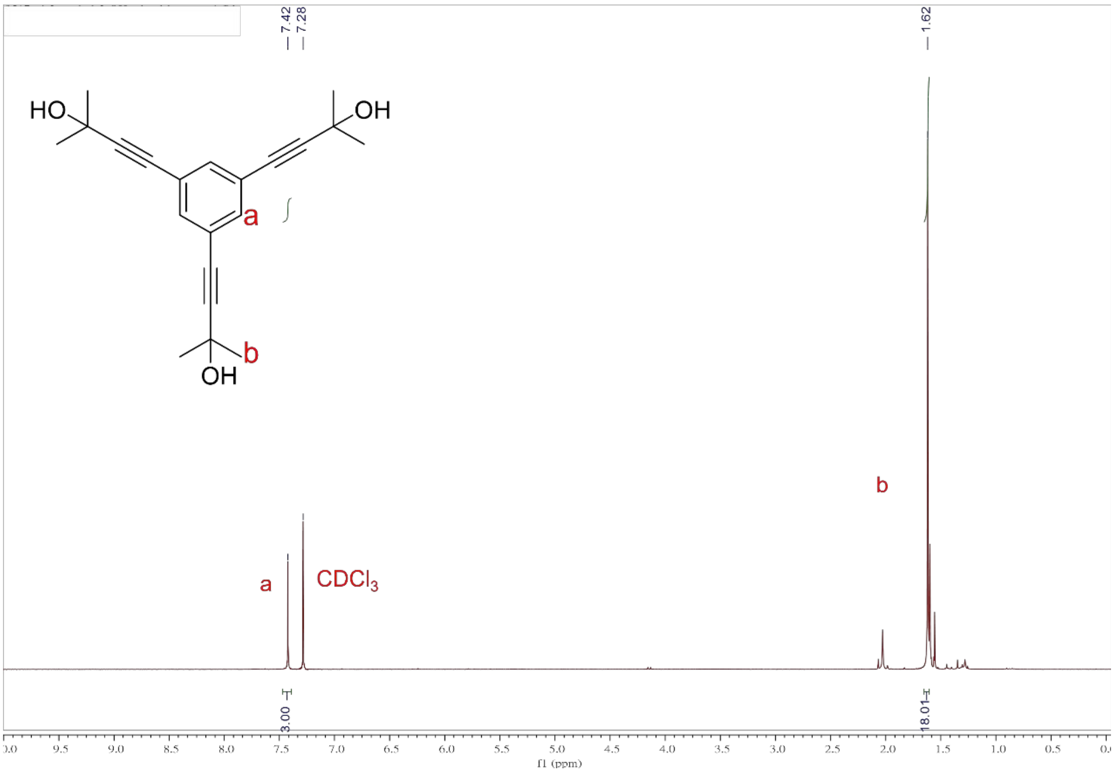

Figure S4. ^{1}H NMR spectrum of 1,3,5-tris(3-methyl-3-hydroxybut-1-ynyl)benzene.

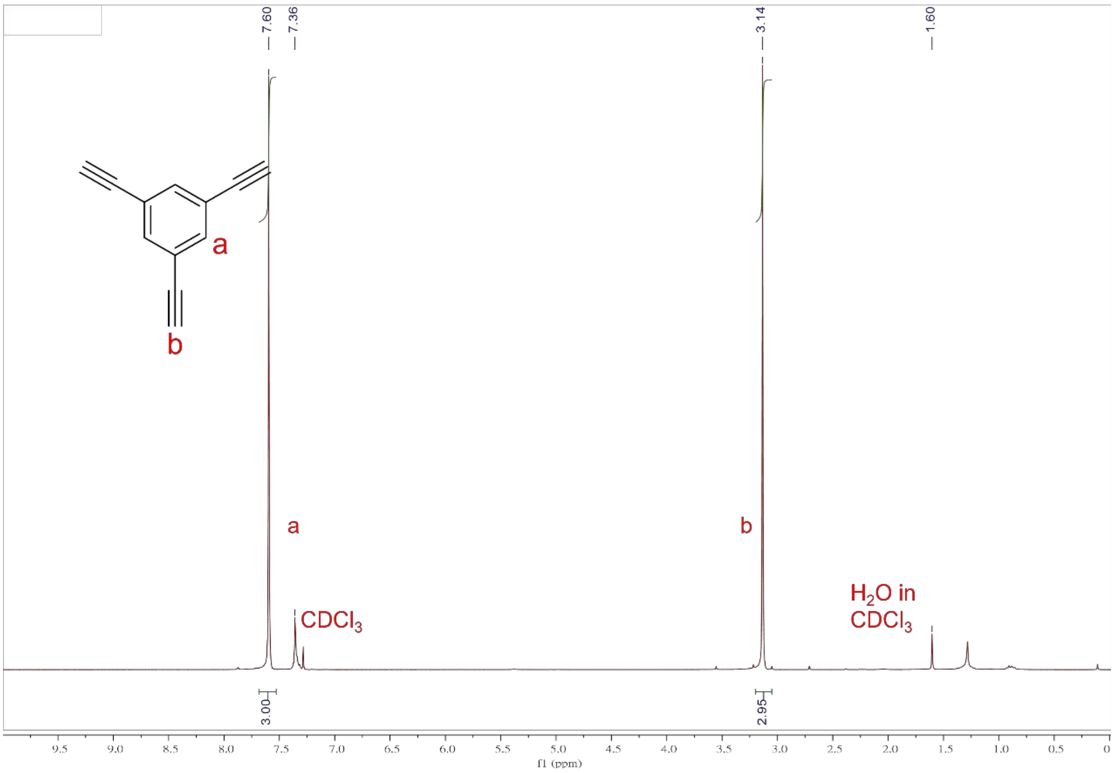

Figure S5. ^{1}H NMR spectrum of 1,3,5-triethynylbenzene.

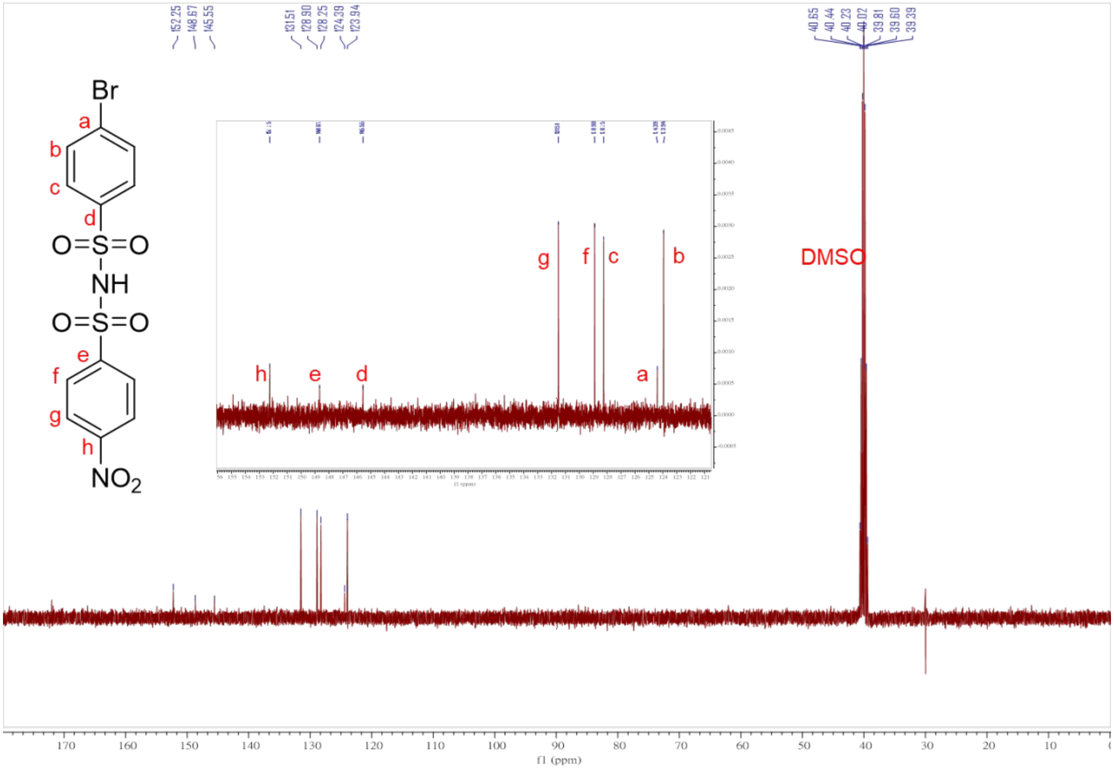

Figure S6. ^{13}C NMR spectrum of BNBSA.

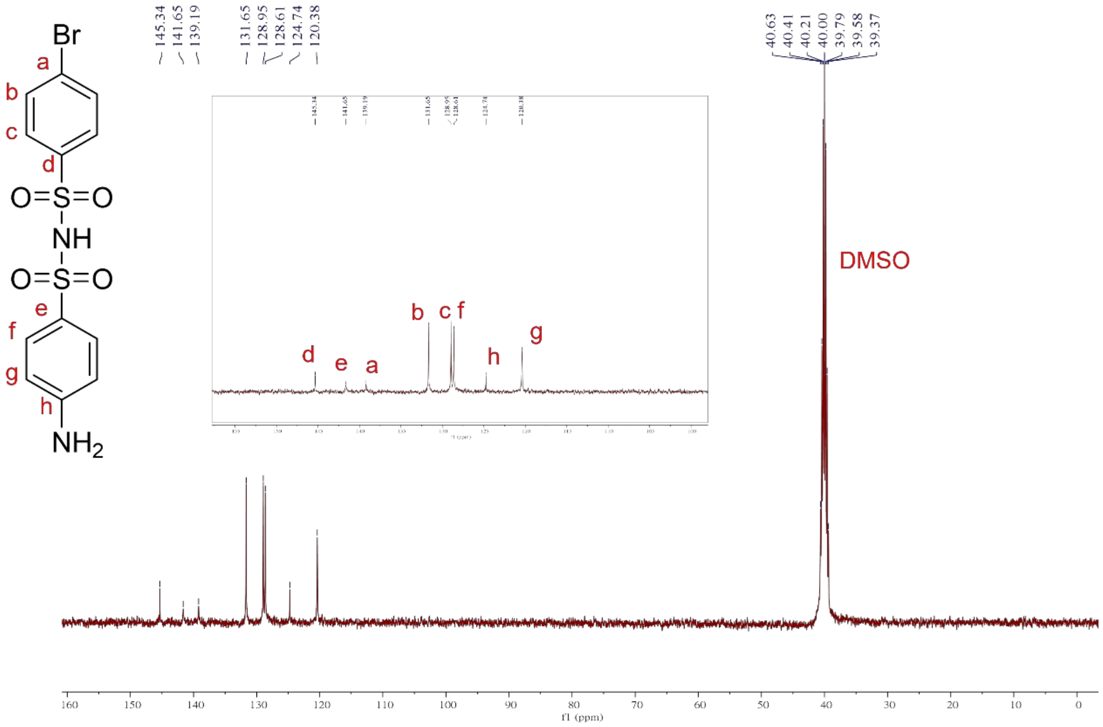

Figure S7. ^{13}C NMR spectrum of BABSA.

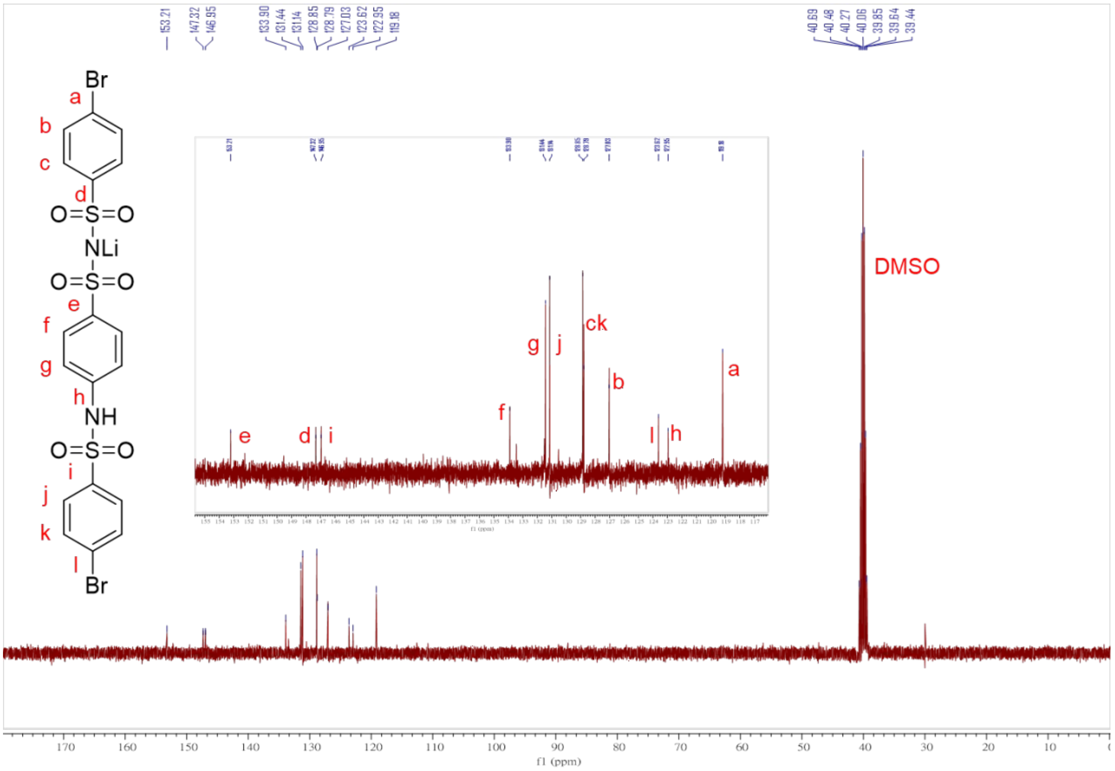

Figure S8. ^{1}H NMR spectrum of Li-BPSSA.

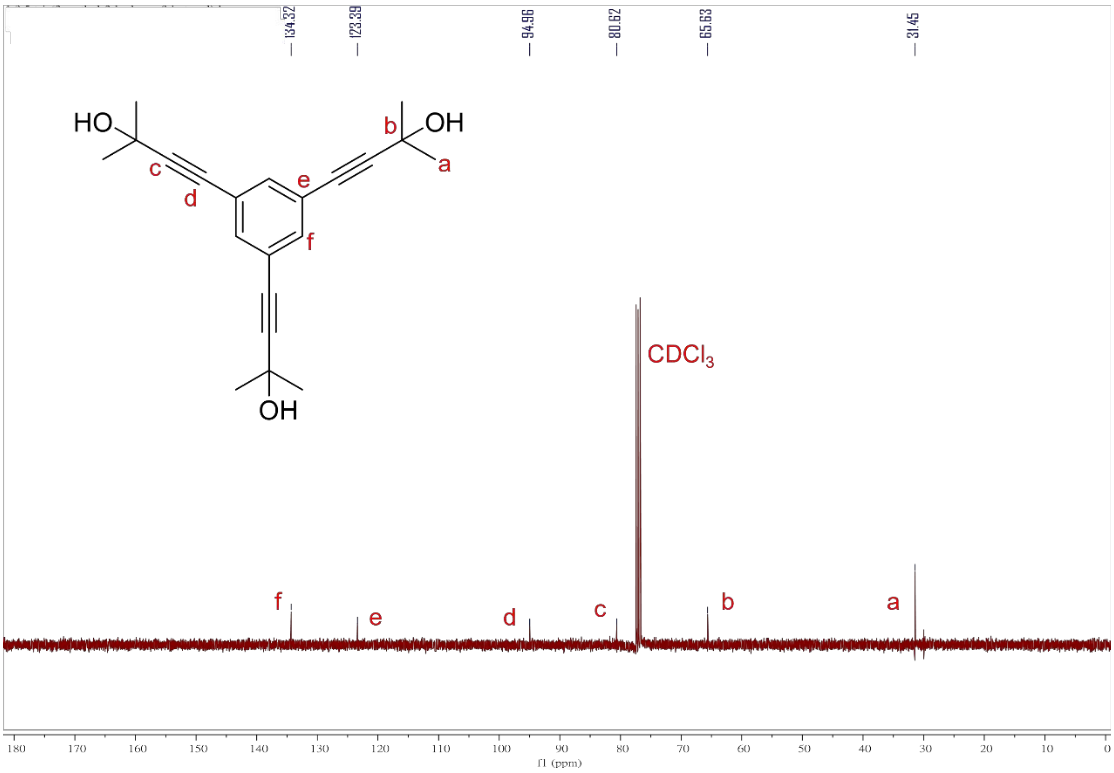

Figure S9. ^{1}H NMR spectrum of 1,3,5-tris(3-methyl-3-hydroxybut-1-ynyl)benzene.

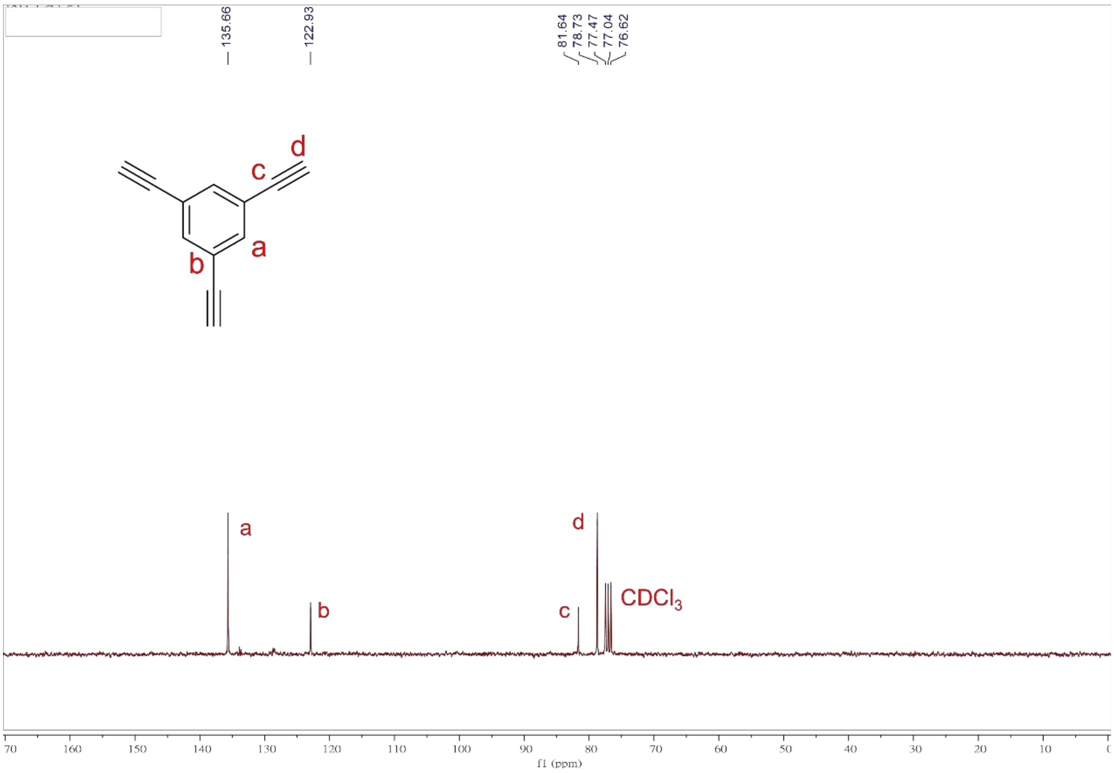

Figure S10. ^{1}H NMR spectrum of 1,3,5-triethynylbenzene.

D:\data\yu\BNBSA_20241224042047.raw 12/24/2024 6:05:18 PM

BNBSA Orbitrap Exploris 120 Negative

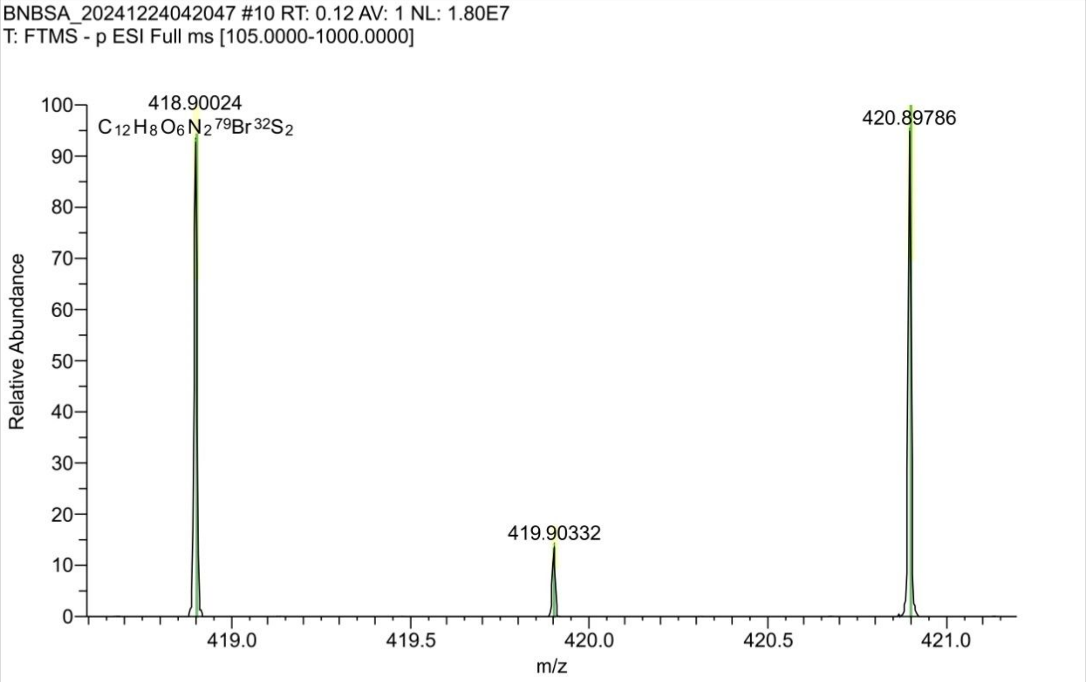

Peak Mass	Display Formula	S Fit	RDB	Delta [ppm]	Theo. mass	Pattern Cov. [%]	MSMS Matched...
418.90024	$C_{12}H_8O_6N_2{}^{79}Br^{32}S_2$	34.3857198732275	9.50	-2.45	418.90126	96.11	(Collection)

Figure S11. HRMS spectrum of BNBSA.

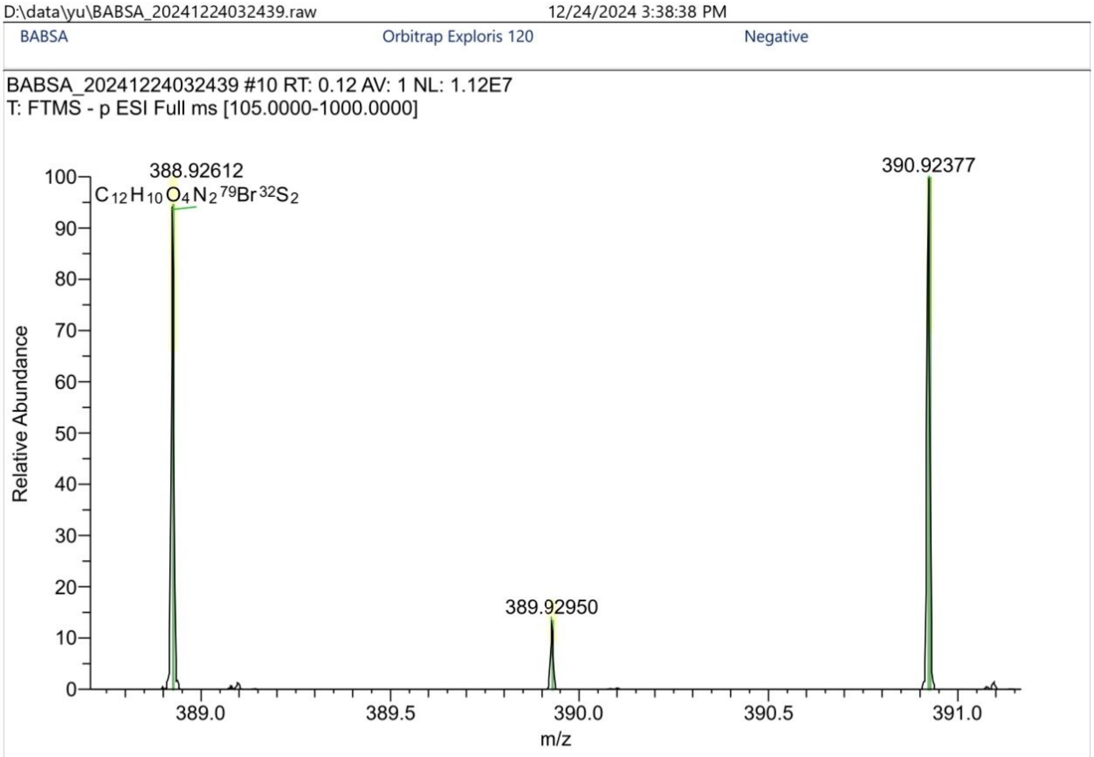

Peak Mass	Display Formula	S Fit	RDB	Delta [ppm]	Theo. mass	Pattern Cov. [%]	MSMS Matched...
388.92612	$C_{12}H_{10}O_4N_2{}^{79}Br^{3}$ ${}^{2}S_2$	37.41532767790 98	8.50	-2.49	388.92708	96.16	(Collection)

Figure S12. HRMS spectrum of BABSA.

D:\data\yu\BPSSA_20241224022338.raw 12/24/2024 2:44:51 PM

BPSSA Orbitrap Exploris 120 Negative

BPSSA_20241224022338 #8 RT: 0.10 AV: 1 NL: 1.85E7
T: FTMS - p ESI Full ms [105.0000-1000.0000]

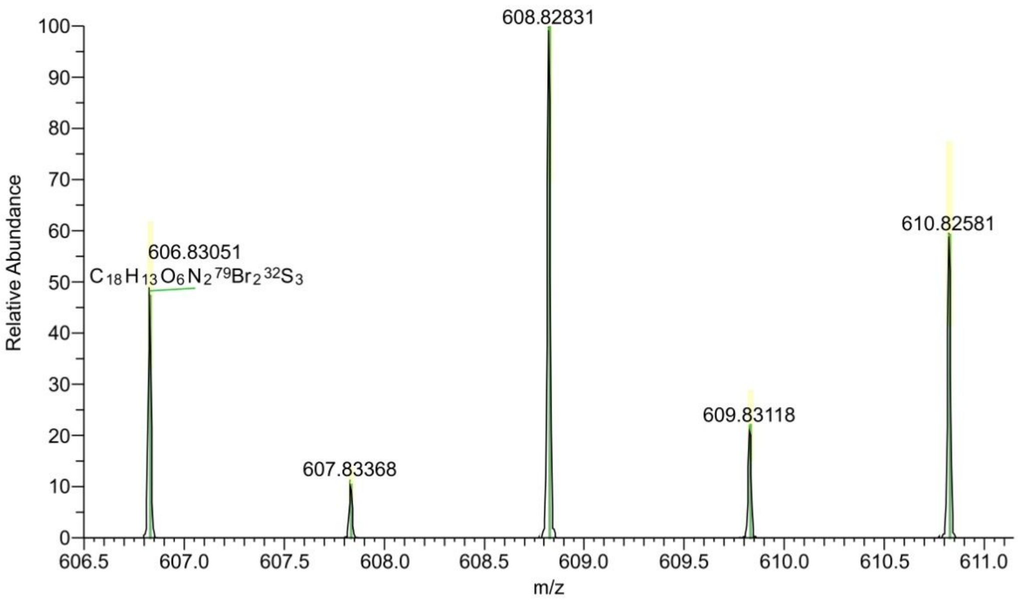

Peak Mass	Display Formula	S Fit	RDB	Delta [ppm]	Theo. mass	Pattern Cov. [%]	MSMS Matched...
606.83051	$C_{18}H_{13}O_6N_2{}^{79}Br_2{}^{32}S_3$	79.429937859315	12.50	-0.48	606.83080	100	(Collection)

Figure S13. HRMS spectrum of BPSSA.

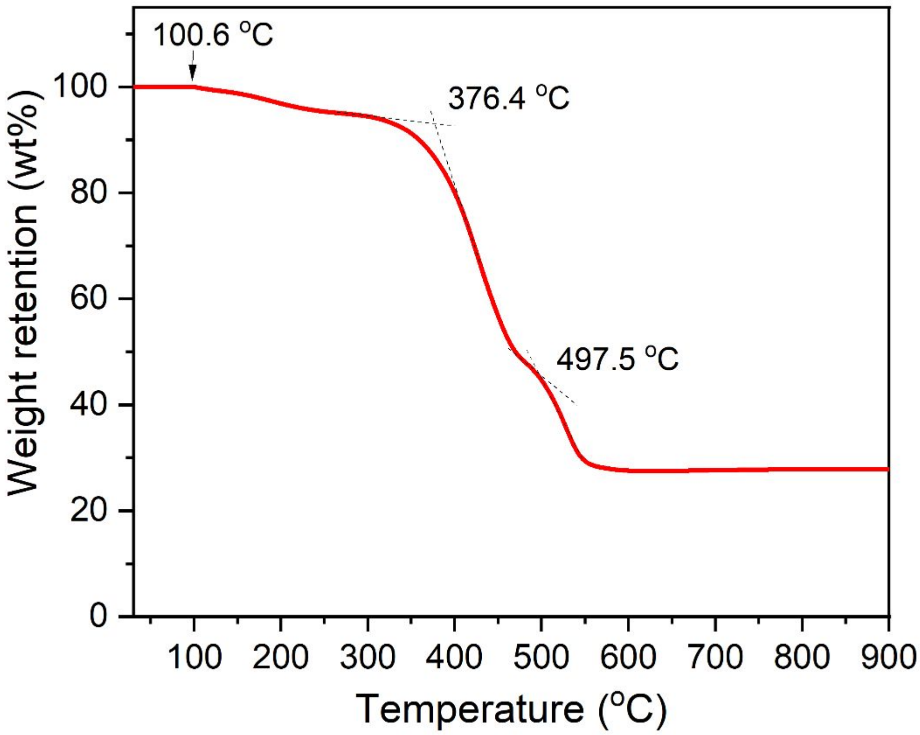

Figure S14. TGA curve of Li-SSP.

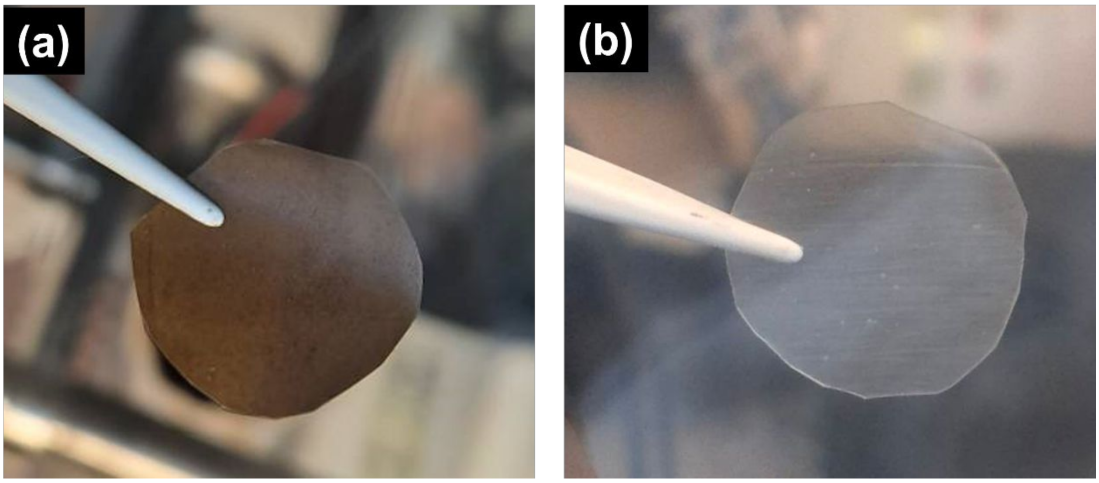

Figure S15. Photographs of the solid-state electrolytes: (a) LiTFSI/PVDF-HFP/Li-SSP and (b) LiTFSI/PVDF-HFP.

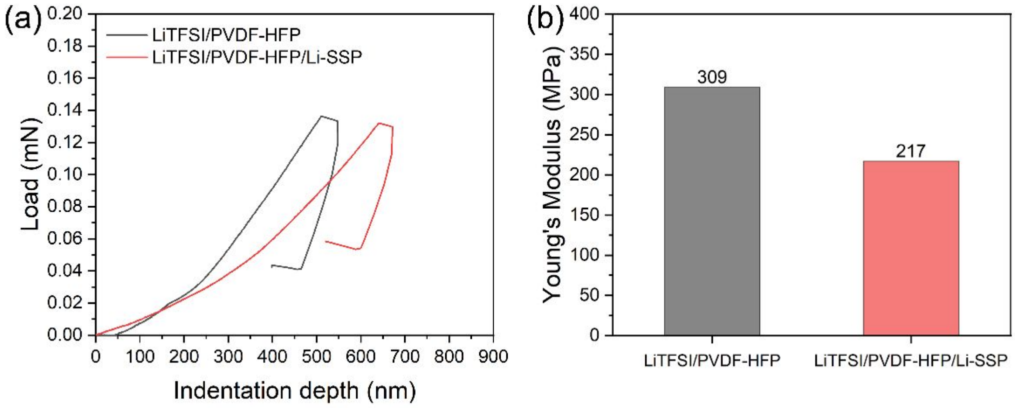

Figure S16. (a) Nanoindentation experimental force-indentation depth and (b) Young's modulus of LiTFSI/PVDF-HFP and LiTFSI/PVDF-HFP/Li-SSP electrolytes.

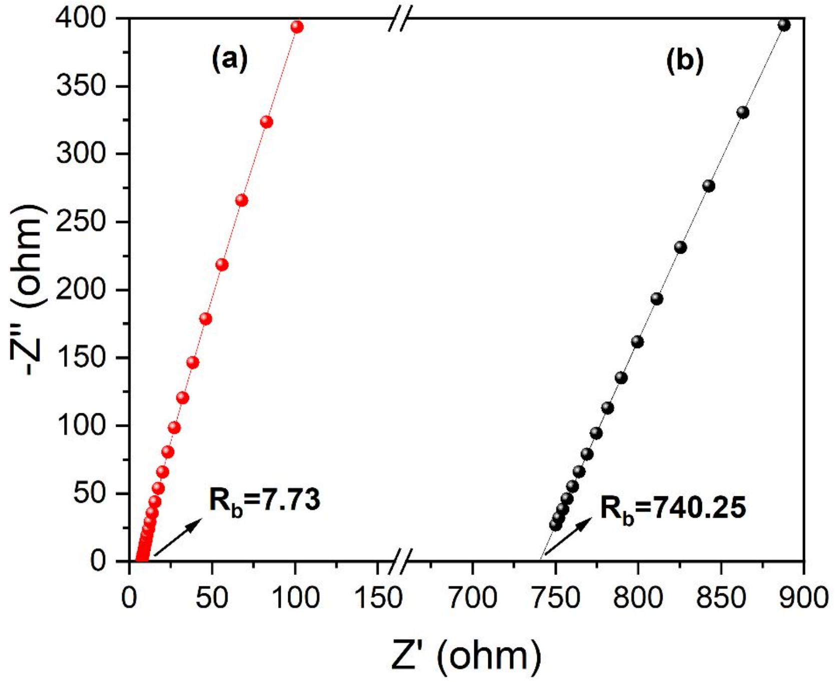

Figure S17 Nyquist plots of (a) SS|Li-SSP composite electrolyte|SS and SS|LiTFSI/PVDF-HFP electrolyte|SS cells at 30 °C.

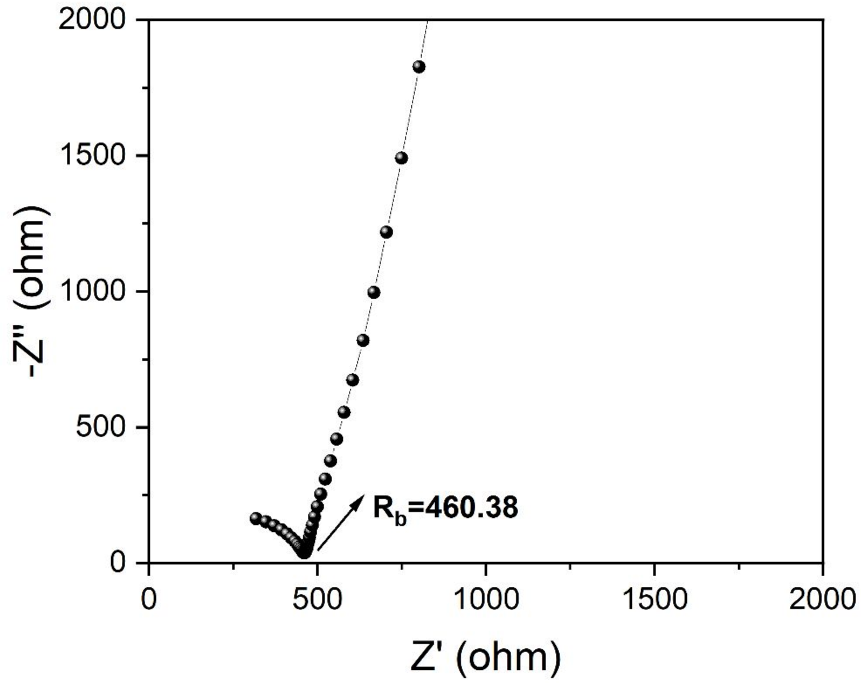

Figure S18 Nyquist plot of SS|PVDF-HFP/Li-SSP|SS cell at 30 °C.

Table S1 Resistance, thickness, area, and ionic conductivity of electrolytes.

Electrolyte	Resistance (ohm)	Thickness (μm)	Area (cm^2)	Ionic conductivity (S cm^{-2})
LiTFSI/PVDF-HFP	740.25	80	2.56	4.22×10^{-6}
LiTFSI/PVDF-HFP/Li-SSP	7.73	80	2.56	4.04×10^{-4}
PVDF-HFP/Li-SSP	460.38	37	2.56	3.14×10^{-6}

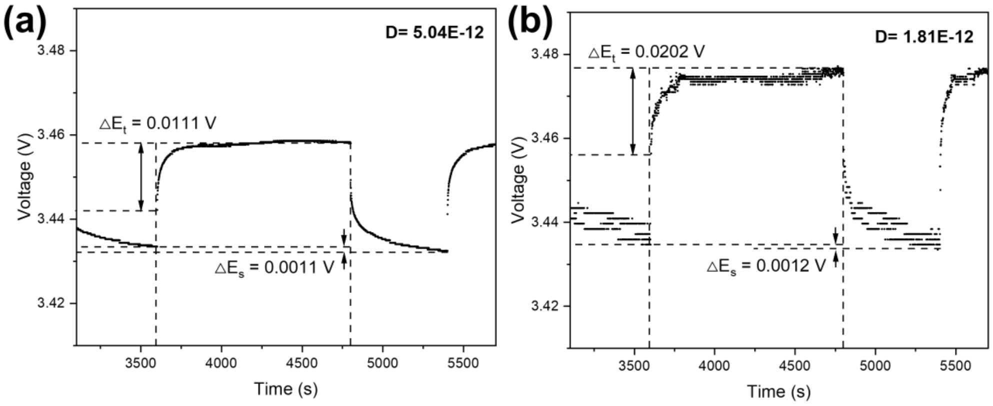

Figure S19. GITT profiles showing the determination of ΔE_t and ΔE_s values for (a) Li|LiTFSI/PVDF-HFP|LFP and (b) Li|LiTFSI/PVDF-HFP/Li-SSP|LFP cells.

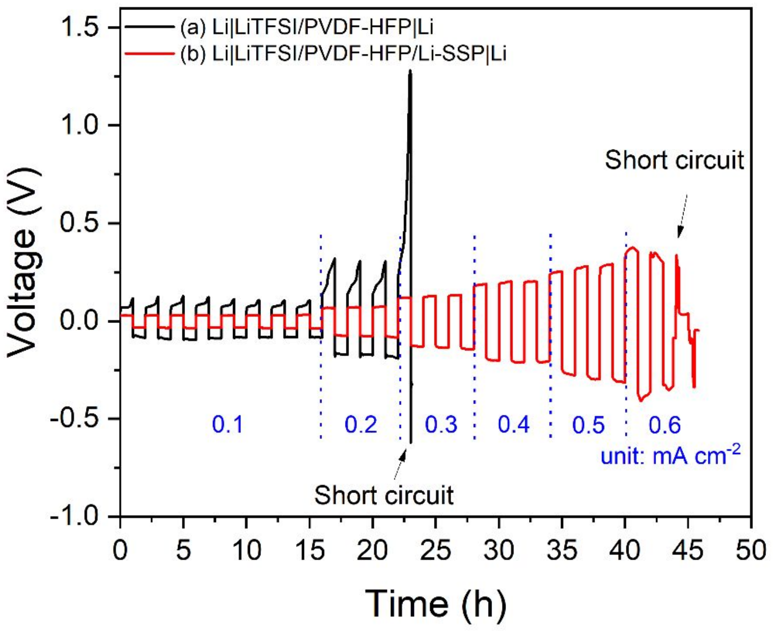

Figure S20. Critical current density measurements of (a) Li|LiTFSI/PVDF-HFP|Li and (b) Li|LiTFSI/PVDF-HFP/Li-SSP|Li cells.

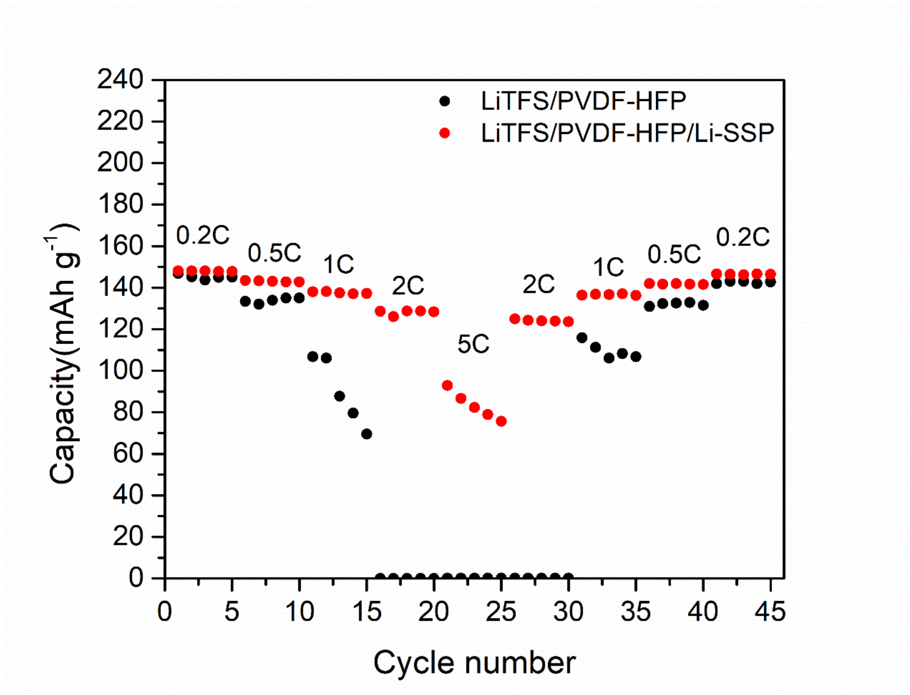

Figure S21. Cycle profiles of Li|LiTFSI/PVDF-HFP|LFP and Li|LiTFSI/PVDF-HFP/Li-SSP|LFP cells at different C-rates.

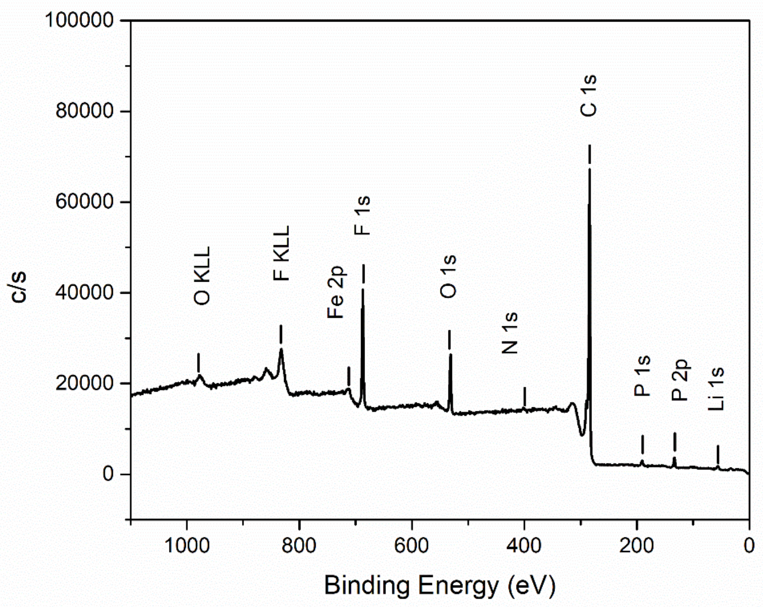

Figure S22. XPS survey spectrum of pristine LFP electrode.

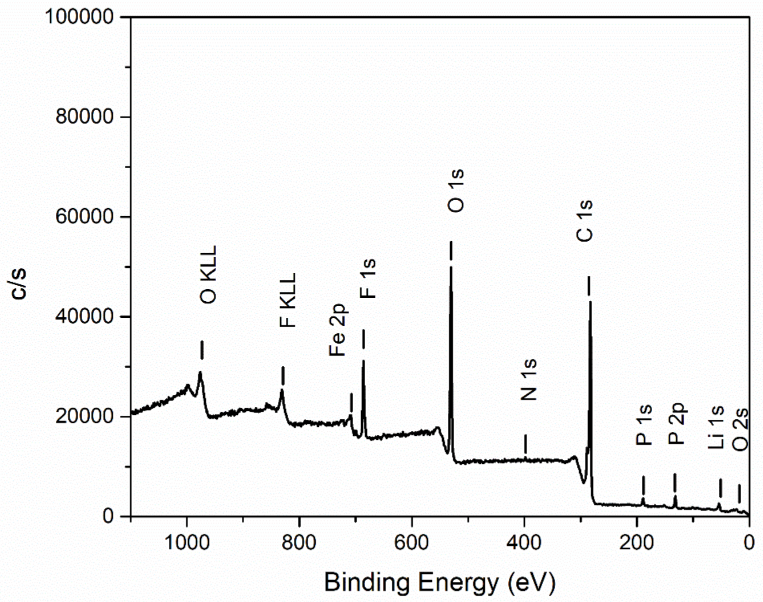

Figure S23. XPS survey spectrum of the LFP electrode after 200 cycles in the LiTFSI/PVDF-HFP electrolyte.

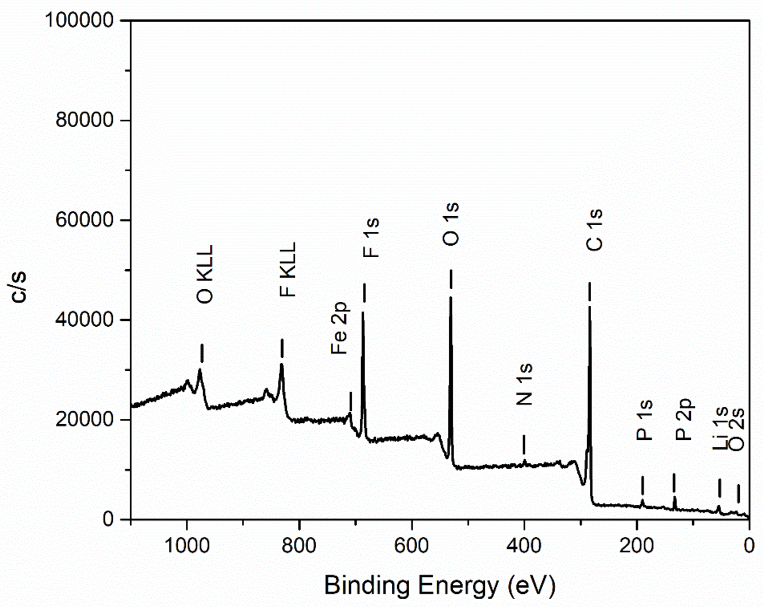

Figure S24. XPS survey spectrum of the LFP electrode after 200 cycles in the LiTFSI/PVDF-HFP/Li-SSP electrolyte.
